# Supplementary figures and images for: Modulation of cellular adhesion, contractility, and migration by MiuA: A comprehensive analysis of its biomechanical impact
Source: PLoS One. 2025 Sep 5;20(9):e0330071. doi: 10.1371/journal.pone.0330071 (PMC12412958; doi:10.1371/journal.pone.0330071)

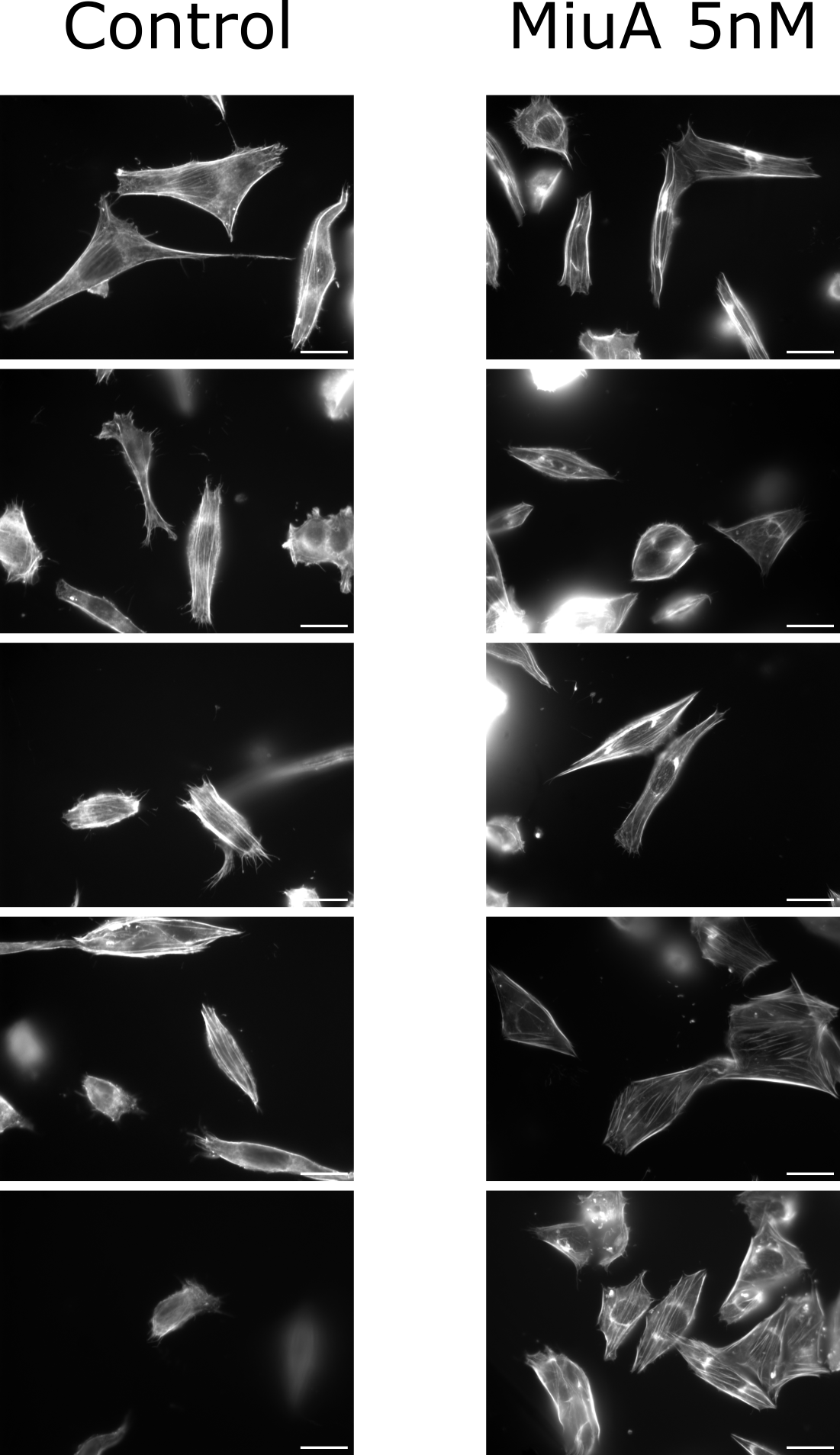

Supplement: S1 Fig — Left: control, right: 5nM MiuA. Scale bar 20µm. (TIF) [file pone.0330071.s001.tif]

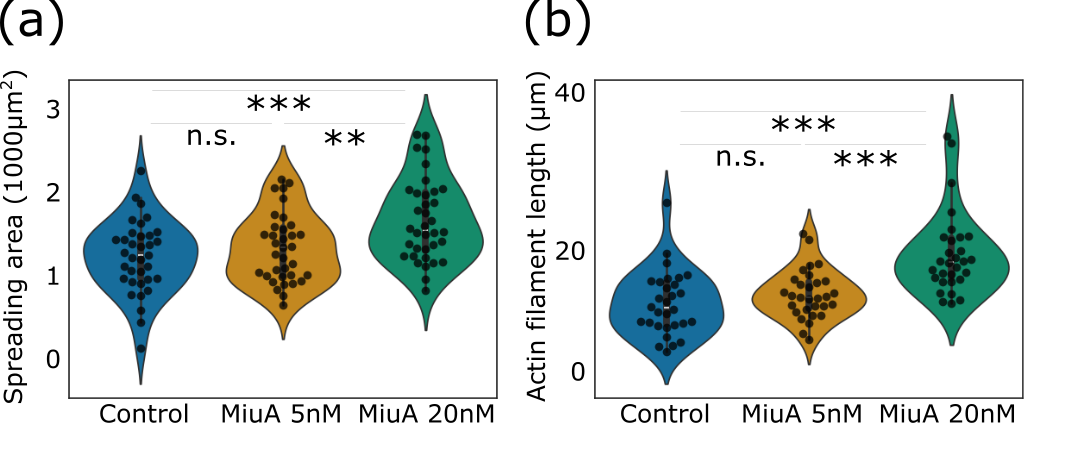

Supplement: S2 Fig — (a) Spreading area of RPE-1 wt cells on a fibronectin (25 µg/ml) coated glass surface. The treatment with 20nM of MiuA significantly increased the spreading area, whereas 5nM showed no significant effect. (b) Mean actin filament length per cell in RPE-1 wt cells. Treatment with 20nM MiuA significantly increased the mean actin filament length, while 5nM showed no significant effect. Number of cells: 35 (a), 30 (b). A statistical analysis was performed using Student’s t test. n.s.: p > 0.05; *: p < 0.05; **: p < 0.01; and ***: p < 0.005. (TIF) [file pone.0330071.s002.tif]

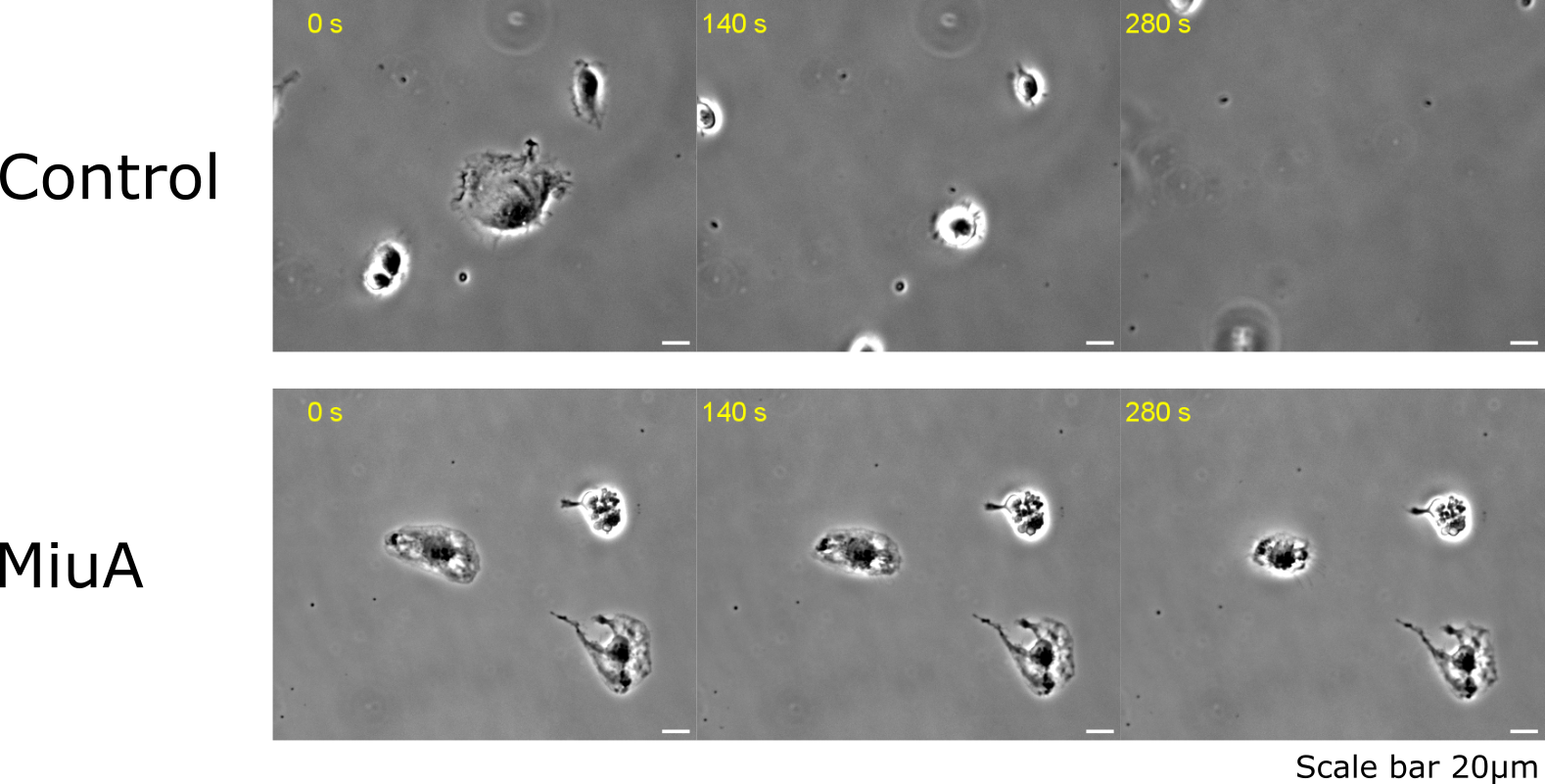

Supplement: S3 Fig — Fully spread RPE-1 LifeAct mCherry cells were trypsinized while being monitored with a video microscope. Pictures were taken every 20 seconds where the time stamp (yellow label in the upper left corner) denotes the time after adding trypsin. The first picture “0 s” was taken immediately after trypsin was added. Top: Control cells treated with DMSO. Bottom: MiuA (20 nM) treated cells. Scale bar 20 μm. (TIF) [file pone.0330071.s003.tif]

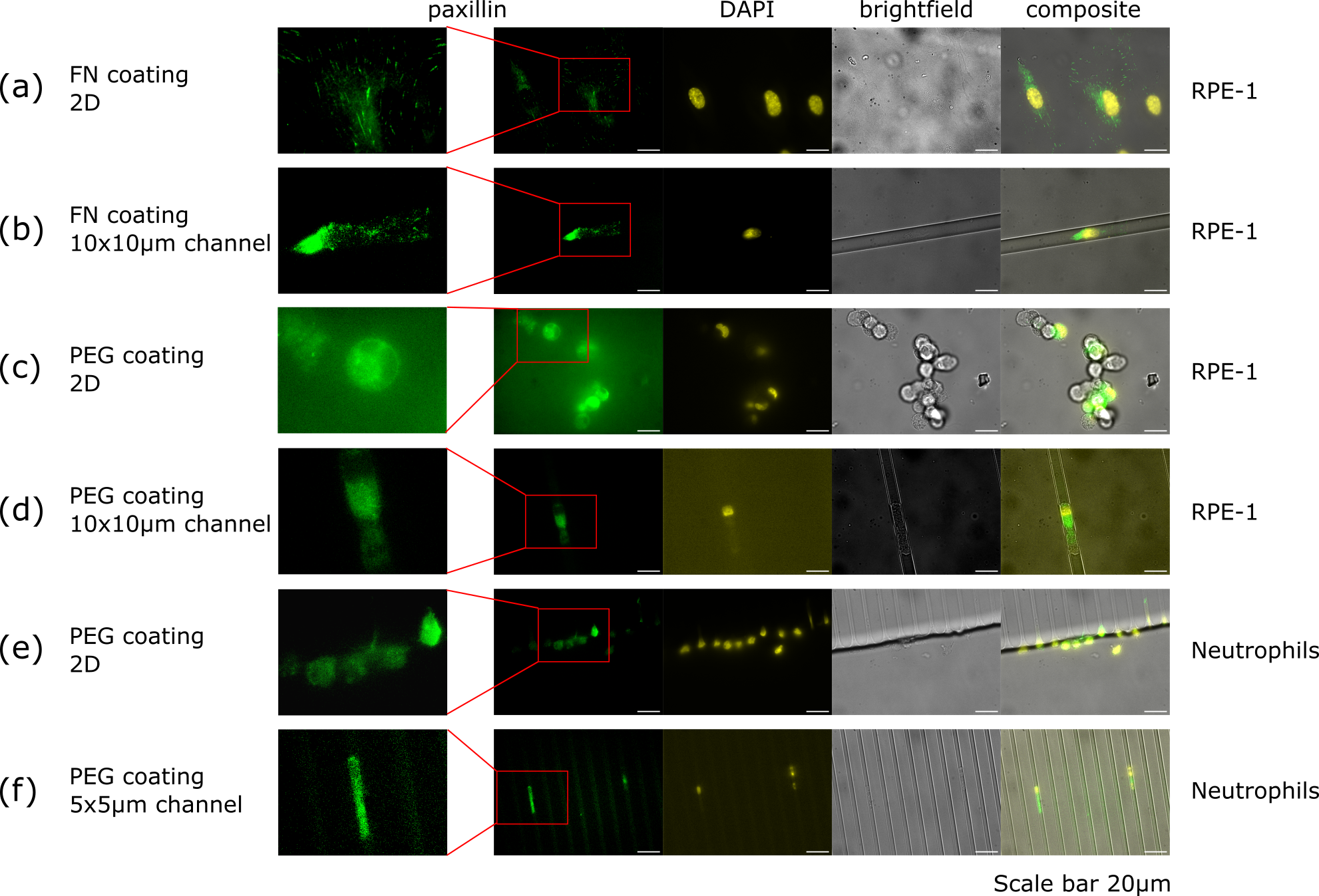

Supplement: S4 Fig — From left to right: Paxillin immunostaining with zoomed in area (red square) in green. DAPI staining of the nucleus in yellow. Brightfield images of RPE-1 cells and neutrophils. Composite images of the paxillin, nucleus and brightfield images. From Top to bottom: (a) RPE-1 cells on 2D fibronectin (25 μg/ml) coated surface. (b) RPE-1 cells inside 10x10 μm PDMS microchannels with fibronectin coating. (c) RPE-1 cells on a PEG-coated surface. (d) RPE-1 cells in PEG-coated 10x10 μm PDMS microchannels. (e) Neutrophils on a PEG-coated 2D surface. (f) Neutrophils inside a PEG-coated 5x5 μm PDMS microchannel. Scale bars: 20 μm. (TIF) [file pone.0330071.s004.tif]

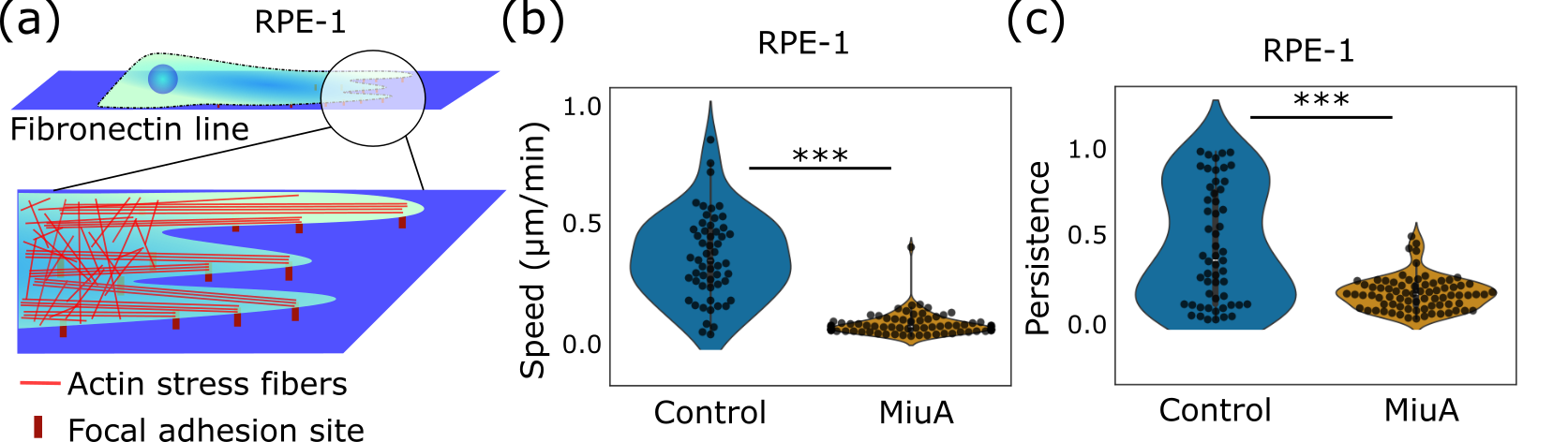

Supplement: S5 Fig — Schematic representation of a mesenchymal migrating cell on a fibronectin line (a). On these fibronectin lines, RPE-1 LifeAct mCherry cells treated with 20 nM MiuA reduced their speed (b) and persistence (c). A statistical analysis was performed using Student’s t test: n.s.: p > 0.05; *: p < 0.05; **: p < 0.01; and ***: p < 0.005. Number of cells: 55 control and 77 MiuA. Data replotted from Baltes et al. [23]. (TIF) [file pone.0330071.s005.tif]
