# Supplementary material for: Modulation of cellular adhesion, contractility, and migration by MiuA: A comprehensive analysis of its biomechanical impact
Source: PLoS One. 2025 Sep 5;20(9):e0330071. doi: 10.1371/journal.pone.0330071 (PMC12412958; doi:10.1371/journal.pone.0330071)
Supplement: S3 File — (PDF) [file pone.0330071.s008.pdf]

Strain energy of cells on patterns depend only on the change of pattern area

1 Introduction

In this protocol I am going to go through most of the calculation steps needed for the analysis of cells, placed on deformable patterns. The idea of this technique origins from Ajinka Ghagre et al (doi: `https://doi.org/10.1021/acsam.1c02987`), where they showed that it is possible to calculate the strain energy of a cell, by measuring the deformation of a underlying pattern without using fluorescent beads, as one would need in traction force microscopy. However, they only use Cartesian coordinates and propose an approximation on how the change in area correlates with the strain energy of cells. We wanted to further advance this technique by transforming the Problem in polar coordinates. There we can calculate the strain energy depending only on the change of the radius of a circle, which then allows us to directly calculate the change of area, resulting on a formula that puts strain energy and area together.

2 Background

As the authors state, most of this and the following section will be taken from "Theory of elasticity 3rd Edition" from Landau and Lifshitz. In section 4 I will start to display what we added to the technique and how we proceeded in polar coordinates.

We start with defining our **displacement vector**:

$$\vec{u} = \begin{pmatrix} u_x \\ u_y \\ u_z \end{pmatrix} = u_x\vec{e}_x + u_y\vec{e}_y + u_z\vec{e}_z \tag{1}$$

Which involves the position of each point after and before displacement

$$u_i = x' - x$$

Definition: Strain Tensor u_{ik}

$$u_{ik} = \frac{1}{2}\left(\frac{\partial u_i}{\partial x_k} + \frac{\partial u_k}{\partial x_i} + \frac{\partial u_l}{\partial x_i} \cdot \frac{\partial u_l}{\partial x_k}\right) \tag{2}$$

Where:

- $i, k, l \in 1, 2, 3$
- $x_1 = x$, $x_2 = y$ and $x_3 = z$
- $\sum_{i=1}^{3} a_i \cdot b_i = a_i \cdot b_i$ (Einstein Notation)

Definition: Stress Tensor σ_{ik}

$$\oint \sigma_{ik} df_k = \int \frac{\partial \sigma_{ik}}{\partial x_k} dV = \int F_i dV \qquad | \qquad F_i = \frac{\partial \sigma_{ik}}{\partial x_k} \tag{3}$$

If we want to display the stress tensor in terms of the strain tensor it will look like this:

$$\sigma_{ik} = \frac{E}{1+\sigma}\left(u_{ik} + \frac{\sigma}{1-2\sigma}u_{ll}\delta_{ik}\right) \tag{4}$$

With:

- E = Young's Modulus
- σ = Poisson ratio
- $\delta_{ik} = 1$ if $i = k$, $\delta_{ik} = 0$ else

Assuming that at equilibrium there are no internal forces (e.g. the sum of all forces is zero) and ignoring gravity, we can say:

$$\vec{F_i} = \frac{\partial \sigma_{ik}}{\partial x_k} = 0 \tag{5}$$

Using the relationship between σ_{ik} and u_{ik} we get:

$$\frac{\partial \sigma_{ik}}{\partial x_k} = \frac{E\sigma}{(a+\sigma)(1-2\sigma)}\frac{\partial u_{ll}}{\partial x_i} + \frac{E}{1+\sigma}\frac{\partial u_{ik}}{\partial x_k} \tag{6}$$

Using also:

$$u_{ik} = \frac{1}{2}\left(\frac{\partial u_i}{\partial x_k} + \frac{\partial u_k}{\partial x_i} + \underbrace{\frac{\partial u_l}{\partial x_k}\frac{\partial u_l}{\partial x_i}}_{=0\text{, as we assume small deformations}}\right) \tag{7}$$

All together we have:

$$\frac{E}{2\cdot(1+\sigma)}\frac{\partial^2}{u\ _i}\partial x_k^2 + \frac{E}{2\cdot(1+\sigma)\cdot(1-2\sigma)}\frac{\partial^2 u_l}{\partial x_i \partial x_l} = 0 \tag{8}$$

Rearranging that equation and using the following equations leaves us with a homogeneous partial differential equation

$$\frac{\partial^2 u_i}{\partial x_k^2} = \Delta\vec{u} \qquad , \qquad \frac{\partial u_l}{\partial x_l} = \vec{\nabla}\cdot\vec{u} \tag{9}$$

$$(1-2\sigma)\Delta\vec{u} + \vec{\nabla}(\vec{\nabla}\cdot\vec{u}) = 0 \tag{10}$$

3 Solving the PDE

In our case we have to deal with the following form of the PDE

$$(1-2\sigma)\Delta\vec{u} + \vec{\nabla}(\vec{\nabla}\cdot\vec{u}) = 0 \tag{11}$$

The right hand side becomes zero as we do not have any intrinsic forces on the body itself, but there are forces applied to the body from the outside that appear in the solution via the boundary conditions. Our solution to the problem will be in the form of

$$\vec{u} = \vec{f} + \vec{\nabla}\phi \tag{12}$$

where $\vec{f}$ satisfies Laplace's equation

$$\Delta\vec{f} = 0$$

and ϕ is some scalar.
If we now substitute (12) into out PDE (11) we obtain:

$$2(1-\phi)\Delta\phi = -\vec{\nabla}\cdot\vec{f} \tag{13}$$

Imagine we only have an elastic plane in the xy plane. That means we can write the x and y components of $\vec{f}$ in the following way:

$$f_x = \frac{\partial g_x}{\partial z} \qquad \text{and} \qquad f_y = \frac{\partial g_y}{\partial z} \tag{14}$$

Since $\vec{f}$ is a harmonic function (meaning it does satisfy the Laplace equation) also its components f_x, f_y do satisfy Laplace equation, and therefore also the functions g_x, g_y:

$$\Delta g_x = \Delta g_y = 0 \tag{15}$$

If we put all of this in our equation (13) we obtain:

$$\Delta\phi = -\frac{1}{2(1-\sigma)}\frac{\partial}{\partial z}\left(\frac{\partial g_x}{\partial x} + \frac{\partial g_y}{\partial y} + f_z\right) \tag{16}$$

As g_x, g_y, f_z are harmonic functions we can rewrite this equation introducing another harmonic function ψ:

$$\phi = -\frac{z}{4(1-\sigma)}\left(\frac{\partial g_x}{\partial x} + \frac{\partial g_y}{\partial y} + f_z\right) + \psi \tag{17}$$

With this the problem reduces to finding the functions g_x, g_y, f_z, ψ which all are harmonic functions that satisfy Laplace equation.

Introducing boundary conditions

As we have a free surface in the xy plane, its corresponding normal vector (surface vector) is in negative z direction. Remembering that

$$\sigma_{ik}\cdot n_k = P_i$$

where P_i denotes the force (pressure) exerted in i direction.
We do no see that in our case we have

$$P_x = -\sigma_{xz}$$
$$P_y = -\sigma_{yz}$$
$$P_z = -\sigma_{zz}$$

If we now use the form of our stress tensor from (4) and express the components of the displacement vector $\vec{u}$ in terms of the quantities f_z, g_x, g_y, ψ we can obtain the following boundary conditions

$$\left[\frac{\partial^2 g_x}{\partial z^2}\right]_{z=0} + \left[\frac{\partial}{\partial x}\left\{\frac{1-2\sigma}{2(1-\sigma)}f_z - \frac{1}{2(1-\sigma)}\left(\frac{\partial g_x}{\partial x} + \frac{\partial g_y}{\partial y}\right) + 2\frac{\partial\psi}{\partial z}\right\}\right]_{Z=0} = -2(1+\sigma)\frac{P_x}{E} \tag{18}$$

$$\left[\frac{\partial^2 g_y}{\partial z^2}\right]_{z=0} + \left[\frac{\partial}{\partial y}\left\{\frac{1-2\sigma}{2(1-\sigma)}f_z - \frac{1}{2(1-\sigma)}\left(\frac{\partial g_x}{\partial x} + \frac{\partial g_y}{\partial y}\right) + 2\frac{\partial\psi}{\partial z}\right\}\right]_{z=0} = -2(1+\sigma)\frac{P_y}{E} \tag{19}$$

$$\left[\frac{\partial}{\partial z}\left\{f_z - \left(\frac{\partial g_x}{\partial x} + \frac{\partial g_y}{\partial y}\right) + 2\frac{\partial\psi}{\partial z}\right\}\right]_{z=0} = -2(1+\sigma)\frac{P_z}{E} \tag{20}$$

Since we have four unknown variable but only three equations to determine them, we can introduce another condition. the authors of "Theory of elasticity 3rd Edition" take the following condition, saying it is proven by the absence of any contradiction in the end result.

$$(1-2\sigma)f_z - \left(\frac{\partial g_x}{\partial x} + \frac{\partial g_y}{\partial y}\right) + 4(1-\sigma)\frac{\partial \psi}{\partial z} = 0 \tag{21}$$

With this additional condition our equations (18) and (19) become

$$\left[\frac{\partial^2 g_x}{\partial z^2}\right]_{z=0} = -\frac{2(1+\sigma)}{E} P_x \tag{22}$$

$$\left[\frac{\partial^2 g_y}{\partial z^2}\right]_{z=0} = -\frac{2(1+\sigma)}{E} P_y \tag{23}$$

Having those equations is all we need to determine the harmonic functions f_z, g_x, g_y, ψ
We also do knwo that harmonic functions that disappear at infinity and have a normal derivative at $z = 0$ are given by the following formula:

$$h(x,y,z) = -\frac{1}{2\pi} \int\int \left[\frac{\partial h(x',y',z)}{\partial z}\right]_{z=0} \frac{1}{r} dx'dy' \tag{24}$$

where $r = \sqrt{(x-x')^2 + (y-y')^2 + z^2}$

If we now seek to calculate all of the components of the vector $\vec{u}$ with regard to the set boundary conditions and setting $z = 0$ we get:

$$u_x = \frac{1+\sigma}{2\pi E r}\left(-\frac{(1-2\sigma)x}{r}F_z + 2(1-\sigma)F_x + \frac{2\sigma x}{r^2}(xF_x + yF_y)\right) \tag{25}$$

$$u_y = \frac{1+\sigma}{2\pi E r}\left(-\frac{(1-2\sigma)y}{r}F_z + 2(1-\sigma)F_y + \frac{2\sigma y}{r^2}(xF_x + yF_y)\right) \tag{26}$$

$$u_z = \frac{1+\sigma}{2\pi E r}\left(2(1-\sigma)F_z + (1-2\sigma)\frac{1}{r}(xF_x + yF_y)\right) \tag{27}$$

Definition: Green Tensor G_{ik}
To solve PDE one can use Green's function which is defined in a way that when put into the PDE, the outcome will be the Dirac delta function:

$$\begin{aligned} L :=& L\left(\frac{d}{dt}\right) = \sum_{k=1}^{N} a_k(t)\frac{d^k}{dt^k} \\ Ly =& f \\ LG(t) =& \delta(t) \\ y(t) =& (G * f)(t) = \int G(t-t') \cdot f(t')dt' \\ Ly(t) =& \int LG(t-t')f(t')dt' = \int \delta(t-t')f(t')dt' = f(t) \end{aligned}$$

So by using Green's idea we can solve our initial PDE by rewriting $\vec{u}$ the following way:

$$\vec{u} = G_{ik} * \vec{F} \tag{28}$$

Where G_{ik} is the **Green Tensor**.
We can extract the Green tensor fro our previous set of equations for the displacement vector $\vec{u}$:

$$G = \frac{1+\sigma}{2\pi E r} \begin{pmatrix} 2(1-\sigma) + \frac{2\sigma x^2}{r^2} & \frac{2\sigma xy}{r^2} & -\frac{(1-\sigma)x}{r} \\ \frac{2\sigma xy}{r^2} & 2(1-\sigma) + \frac{2\sigma y^2}{r^2} & -\frac{(1-\sigma)y}{r} \\ \frac{1-2\sigma}{r}x & \frac{1-\sigma}{r}y & 2(1-\sigma) \end{pmatrix}$$

As we are only interested in the deformation in x-y direction, we can neglect all components regarding z, leaving us with:

$$G = \frac{1+\sigma}{2\pi E r} \begin{pmatrix} 2(1-\sigma) + \frac{2\sigma x^2}{r^2} & \frac{2\sigma xy}{r^2} \\ \frac{2\sigma xy}{r^2} & 2(1-\sigma) + \frac{2\sigma y^2}{r^2} \end{pmatrix} \tag{29}$$

In our case we usually obtain the displacement fields and are interested in the force that was necessary to deform our plane in such a way. For this, we need to inverse equation (28), which is difficult, as we do not have a simple Matrix multiplication but a convolution of a matrix and a vector.
To facilitate this, we can perform a Fourier transformation so that the convolution would change into a multiplication, which we easily can inverse. Afterwards performing the inverse Fourier transformation results in an equation we can use to calculate the forces that were needed for our observed displacement

$$\vec{u} = G * \vec{F} \quad \textbf{Fourier} \ \rightarrow \hat{\vec{u}} = \hat{G} \cdot \hat{\vec{F}} \quad \textbf{Inverting} \ \rightarrow \hat{\vec{F}} = \hat{G}^{-1} \cdot \hat{\vec{u}} \quad \textbf{reverse Fourier} \ \rightarrow \vec{F} = G^{-1} * \vec{u}$$

4 Polar coordinates

Ajinka Ghagre et al (doi: `https://doi.org/10.1021/acsam.1c02987`) managed to write a Matlab script that performs this transformations in Cartesian coordinates. They simulate a square grid which starts with the initial area and shrinks isotropic into a smaller square grid after deformation. Thus allowing them to calculate the exerted force on the underlying substrate and therefore the strain energy

$$U = \frac{1}{2} \int \vec{f} \cdot \vec{u} \, dA \tag{30}$$

As in this case we loose all information about the real force and displacement field, we thought about switching from a Cartesian system into a polar system. This would reduce the needed parameters from 2 (x and y) in Cartesian to 1 (r) in polar.
To know whether we can simply transform our Green tensor, we want to derive equation 11 by assuming polar coordinates from the very beginning.
In our case we want to use cylindrical coordinates as we are going to solve our equation solely in the x-y-plane (or now in the r-φ-plane). For this we have:

$$x = r \cdot cos(\varphi)$$
$$y = r \cdot sin(\varphi)$$
$$z = z$$

And for the derivatives we find:

$$\frac{\partial r}{\partial x} = cos(\varphi)$$
$$\frac{\partial r}{\partial y} = sin(\varphi)$$
$$\frac{\partial \varphi}{\partial x} = -\frac{1}{r} sin(\varphi)$$
$$\frac{\partial \varphi}{\partial y} = \frac{1}{r} cos(\varphi)$$

This leads us to the following relations:

$$\begin{aligned} \frac{\partial}{\partial x} &= \frac{\partial r}{\partial x}\frac{\partial}{\partial r} + \frac{\partial \varphi}{\partial x}\frac{\partial}{\partial \varphi} = cos(\varphi)\frac{\partial}{\partial r} - \frac{sin(\varphi)}{r}\frac{\partial}{\partial \varphi} \\ \frac{\partial}{\partial y} &= \frac{\partial r}{\partial y}\frac{\partial}{\partial r} + \frac{\partial \varphi}{\partial y}\frac{\partial}{\partial \varphi} = sin(\varphi)\frac{\partial}{\partial r} + \frac{cos(\varphi)}{r}\frac{\partial}{\partial \varphi} \end{aligned} \tag{31}$$

We also want to display our displacement vector $\vec{u}$ in those polar coordinates- Looking at FIgure S1 leads us to:

$$u_x = u_r cos(\varphi) - u_\varphi sin(\varphi)$$
$$u_y = u_r sin(\varphi) + u_\varphi cos(\varphi)$$
$$u_z = u_z$$

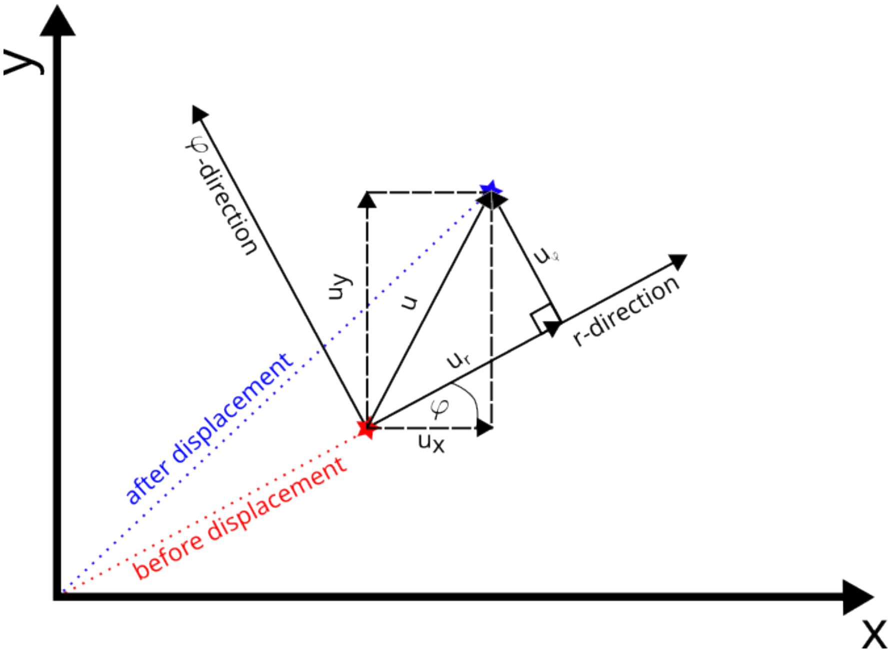

Figure S1: Visual display of the displacement vector in polar coordinates

Our strain tensor in cylindrical coordinates should therefore look as follows:

$$\begin{pmatrix} u_{rr} & u_{r\varphi} & u_{rz} \\ u_{\varphi r} & u_{\varphi\varphi} & u_{\varphi z} \\ u_{zr} & u_{z\varphi} & u_{zz} \end{pmatrix} = \begin{pmatrix} cos(\varphi) & sin(\varphi) & 0 \\ -sin(\varphi) & cos(\varphi) & 0 \\ 0 & 0 & 1 \end{pmatrix} \begin{pmatrix} u_{xx} & u_{xy} & u_{xz} \\ u_{yx} & u_{yy} & u_{yz} \\ u_{zx} & u_{zy} & u_{zz} \end{pmatrix} \begin{pmatrix} cos(\varphi) & -sin(\varphi) & 0 \\ sin(\varphi) & cos(\varphi) & 0 \\ 0 & 0 & 1 \end{pmatrix}$$

If we now put in all we know already, our tensor elements become:

$$\begin{aligned}
u_{rr} &= u_{xx} \cdot cos(\varphi)^2 + u_{yy} \cdot sin(\varphi)^2 + u_{xy} \cdot sin(2\varphi) \\
u_{\varphi\varphi} &= u_{xx} \cdot sin(\varphi)^2 + u_{yy} \cdot cos(\varphi)^2 - u_{xy} \cdot sin(2\varphi) \\
u_{zz} &= u_{zz} \\
u_{r\varphi} &= (u_{yy} - u_{xx}) \cdot cos(\varphi)sin(\varphi) + u_{xy} \cdot (cos(\varphi)^2 - sin(\varphi)^2) \\
u_{rz} &= u_{zx} \cdot cos(\varphi) + u_{zy} \cdot sin(\varphi) \\
u_{z\varphi} &= -u_{zx} \cdot sin(\varphi) + u_{zy} \cdot cos(\varphi)
\end{aligned}$$

Taking now together everything we know from equation (7) and (31), we obtain:

$$\begin{aligned}
u_{rr} &= \frac{\partial u_r}{\partial r} \\
u_{\varphi\varphi} &= \frac{1}{r}\frac{\partial u_\varphi}{\partial \varphi} + \frac{u_r}{r} \\
u_{zz} &= \frac{\partial u_z}{\partial z} \\
u_{r\varphi} &= \frac{1}{2}\left(\frac{1}{r}\frac{\partial u_r}{\partial \varphi} + \frac{\partial u_\varphi}{\partial r} - \frac{u_\varphi}{r}\right) \\
u_{rz} &= \frac{1}{2}\left(\frac{\partial u_r}{\partial z} + \frac{\partial u_z}{\partial r}\right) \\
u_{z\varphi} &= \frac{1}{2}\left(\frac{1}{r}\frac{\partial u_z}{\partial \varphi} + \frac{\partial u_\varphi}{\partial z}\right)
\end{aligned} \tag{32}$$

Using Hook's law:

$$\sigma_{ij} = \frac{E}{(1+\sigma)}\left(u_{ij} + \frac{\sigma}{(1-2\sigma)}u_{kk}\delta_{ij}\right) \tag{33}$$

The definitions of ∇ and Δ in polar coordinates:

$$\begin{aligned}
\vec{\nabla} &= \vec{e}_r\frac{\partial}{\partial r} + \frac{1}{r}\vec{e}_\varphi\frac{\partial}{\partial \varphi} + \vec{e}_z\frac{\partial}{\partial z} \\
\Delta &= \vec{\nabla} \cdot \vec{\nabla}
\end{aligned} \tag{34}$$

and knowing that at equilibrium we have:

$$\vec{\nabla} \cdot \sigma_{\text{Stress Tensor}} = 0 \tag{35}$$

we can put everything together and see, that we still end up with our PDE from above (10):

$$(1-2\sigma)\Delta\vec{u} + \vec{\nabla}\left(\vec{\nabla} \cdot \vec{u}\right) = 0$$

This means, we can solve our equation regardless of the choice of our coordinate system, which means, that one can solve the PDE as described above and transform our solution (Green Tensor) according to the designated coordinate system.
In our case we transformed the Green tensor into polar coordinates (r, φ):

$$G_{\text{polar}} = \frac{1+\sigma}{\pi E r}\begin{pmatrix} (1-\sigma)+\sigma & 0 \\ 0 & (1-\sigma) \end{pmatrix} \qquad | \text{ assuming } \varphi = 0 \tag{36}$$

We also have to transform our displacement vector $\vec{u}$ into polar coordinates (r, φ):

$$\vec{u}(r,\varphi) = \begin{pmatrix} r' - r \\ 0 \end{pmatrix} \tag{37}$$

Now we have to transform both of them with the following Fourier Transformation:

$$Y_{p+1,q+1} = \sum_{j=0}^{N-1} \sum_{k=0}^{M-1} \left(e^{-\frac{2\pi i}{N}}\right)^{jp} \cdot \left(e^{-\frac{2\pi i}{M}}\right)^{kq} \cdot X_{j+1,k+1} \tag{38}$$

Where Y denotes the Fourier transformed variable, X the original variable, N and M the dimensions of the Matrix respectively.
In our case we have a 2×2 Matrix G_{polar} and a 2-dimensional vector $\vec{u}$ which plugged in in equation (38) results in:

$$\hat{G}_{polar} = \frac{1+\sigma}{\pi E r} \begin{pmatrix} 2(1-\sigma)+\sigma & \sigma \\ \sigma & 2(1-\sigma)+\sigma \end{pmatrix} \tag{39}$$

$$\downarrow \text{Inverting}$$

$$\hat{G}_{polar}^{-1} = \frac{\pi E r}{(1+\sigma)[(2(1-\sigma)+\sigma)^2 - \sigma^2]} \begin{pmatrix} 2(1-\sigma)+\sigma & -\sigma \\ -\sigma & 2(1-\sigma)+\sigma \end{pmatrix} \tag{40}$$

$$\hat{\vec{u}} = (r'-r) \begin{pmatrix} 1 \\ 1 \end{pmatrix} \tag{41}$$

Following our idea earlier on, we can now compute the force $\hat{\vec{F}}$ and afterwards perform a reverse Fourier transformation.

$$\hat{\vec{F}} = \frac{2\pi E r (r'-r) 2(1-\sigma)}{(1+\sigma)[(2(1-\sigma)+\sigma)^2 - \sigma^2]} \begin{pmatrix} 1 \\ 1 \end{pmatrix} \tag{42}$$

Using the formula for the reverse Fourier Transformation:

$$X_{p,q} = \frac{1}{M} \sum_{j=1}^{m} \frac{1}{N} \sum_{k=1}^{N} \left(e^{-\frac{2\pi i}{M}}\right)^{(j-1)(p-1)} \left(e^{-\frac{2\pi i}{N}}\right)^{(k-1)(q-1)} Y_{j,k} \tag{43}$$

We get:

$$\vec{F} = \frac{2\pi E r (r'-r) 2(1-\sigma)}{(1+\sigma)[(2(1-\sigma)+\sigma)^2 - \sigma^2]} \begin{pmatrix} 1 \\ 0 \end{pmatrix} \tag{44}$$

5 Strain energy and pattern area

The compute the strain energy, we need the force applied to the substrate per unit of area and the displacement field as described in equation (30) . By dividing our force $\vec{F}$ by the area of the change $\pi(r'-r)^2$, we obtain the needed force $\vec{f}$. This results in the following integral:

$$U = \frac{1}{2} \int \vec{f} \cdot \vec{u} \, dA = \frac{1}{2} \int 2 \underbrace{\frac{E(1-\sigma)}{(1+\sigma)[(2(1-\sigma)+\sigma)^2 - \sigma^2]}}_{:=a} \cdot r \, dA \tag{45}$$

Solving this integral leads us to the following:

$$U = a \int_{r'}^{r_i} \int_0^{2\pi} r^2 dr d\varphi = \frac{2\pi a}{3} \left(r_i^3 - r'^3\right) \quad | \, A = \frac{r^2}{\pi} \tag{46}$$

$$= \frac{2\pi a}{3} \left(\left(\frac{A_i}{\pi}\right)^{\frac{3}{2}} - \left(\frac{A'}{\pi}\right)^{\frac{3}{2}} \right) \tag{47}$$

If we now consider that we reduced our problem to a 1D problem only depending on the radius r, we need to divide this Energy by 2π, leaving us with the final expression:

$$\boxed{U_{\text{final}} = \frac{a}{3}\left(\left(\frac{A_i}{\pi}\right)^{\frac{3}{2}} - \left(\frac{A'}{\pi}\right)^{\frac{3}{2}}\right)} \tag{48}$$

6 Comparison between polar and Cartesian

To check whether our way of calculating the strain energy aligns with the ones from Gharge et al, we plugged in the same set of data for all three algorithms. The results can be seen in Figure S2 and S3.

Especially Figure S3 shows that our way of calculating the strain energy aligns well with the other two. Having a closer look to the values at greater deformation one could also say that our way of calculating the strain energy aligns better with the Cartesian solution of Gharge et al. than their simplified version.

Since both ways (simplified and Cartesian) were proven to fit the experimental datasets, we conclude that our solution does as well fit the data and might well be used to calculate the strain energy in cells deforming an underlying soft pattern.

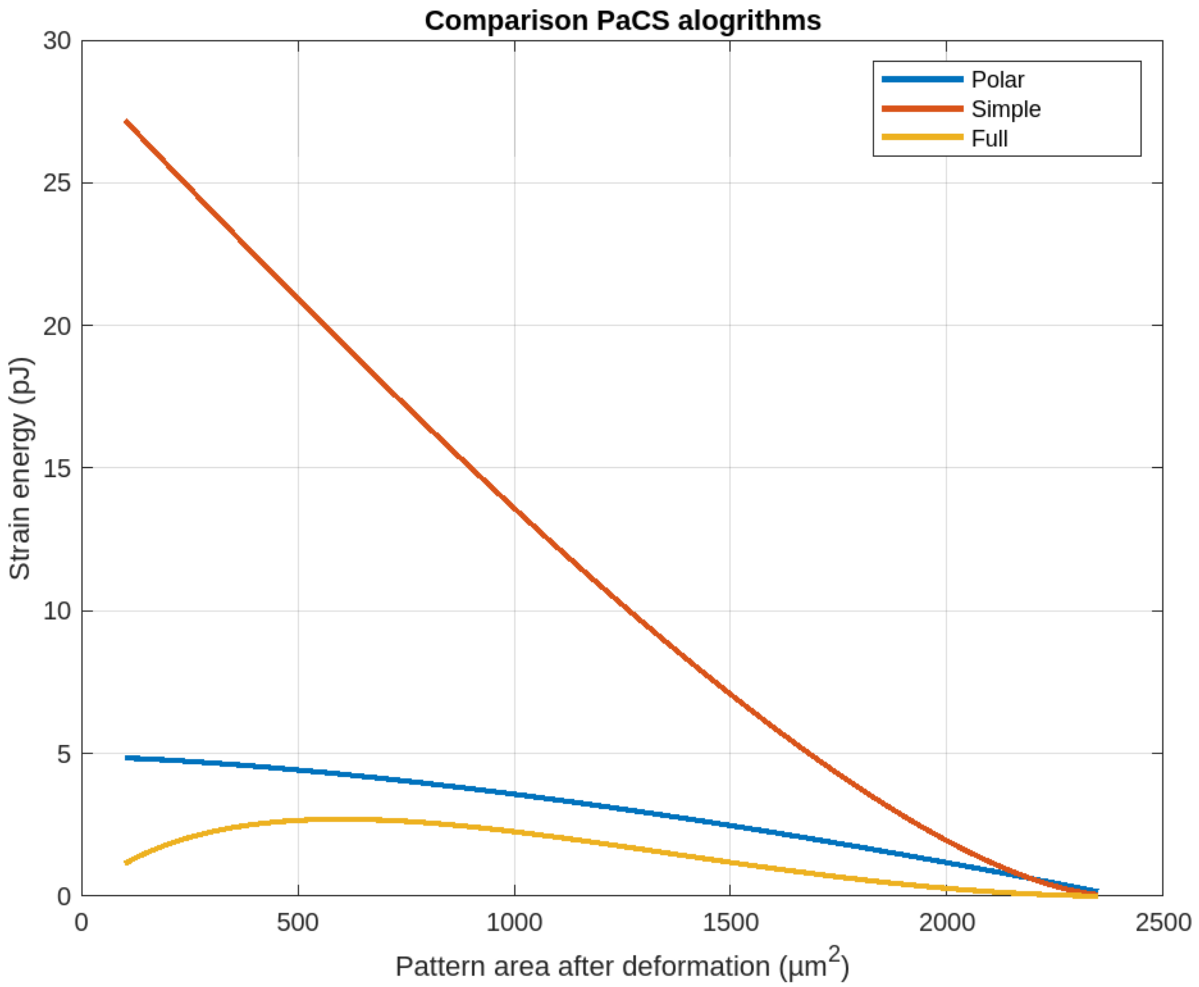

Figure S2: Comparison between the three approaches on calculating the strain energy. (RED) simplified version, (YELLOW) Fourier transformation of mesh-grid in Cartesian coordinates. (BLUE) transformation of Green Tensor in polar coordinates. For all three cases the assumption was ”small deformations”

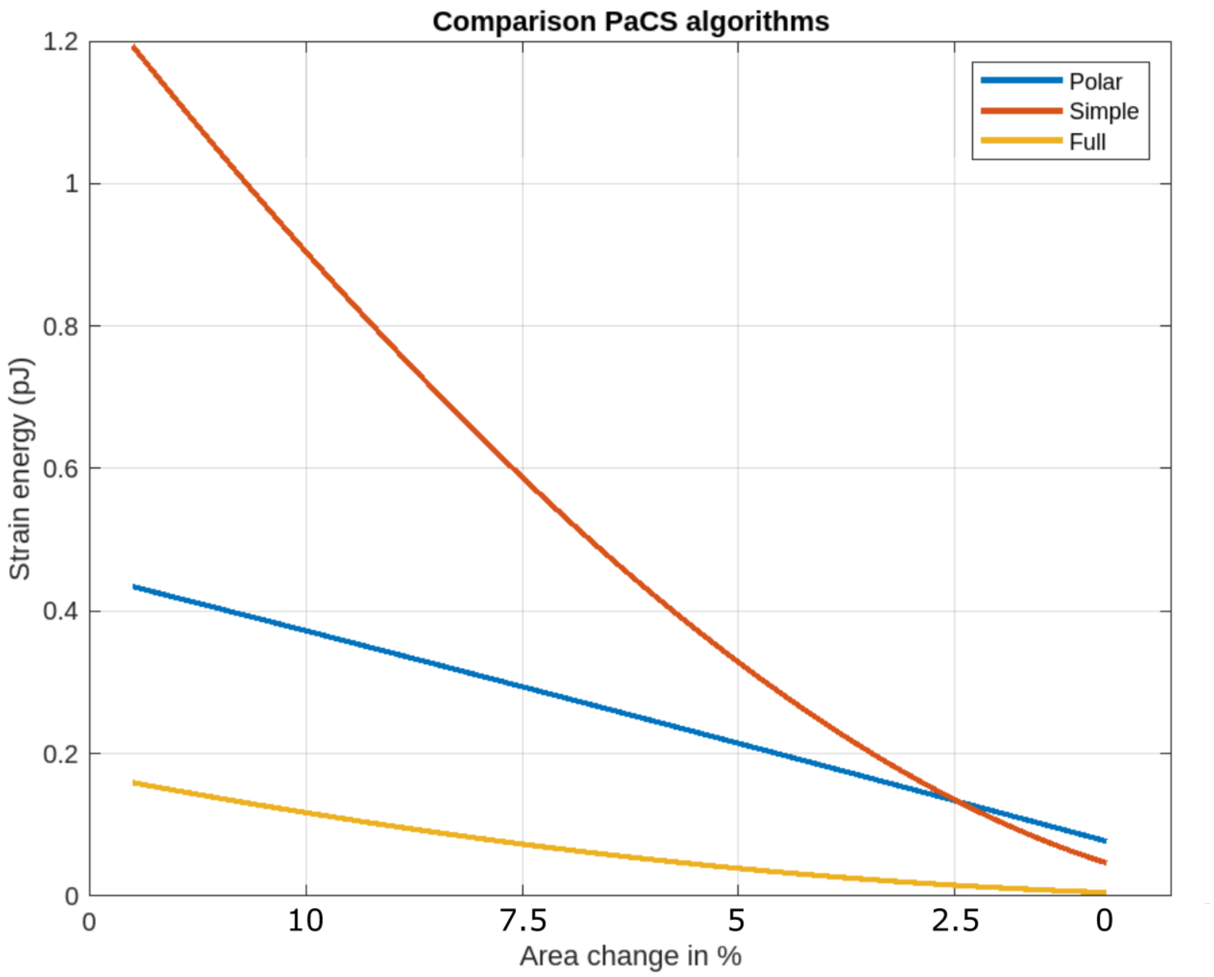

Figure S3: Comparison between the three approaches on calculating the strain Energy, showing only small deformations in the range lower than 15% (RED) simplified version, (YELLOW) Fourier transformation of mesh-grid in Cartesian coordinates. (BLUE) transformation of Green Tensor in polar coordinates. For all three cases the assumption was "small deformations".
