# Supplementary material for: Modulation of cellular adhesion, contractility, and migration by MiuA: A comprehensive analysis of its biomechanical impact
Source: PLoS One. 2025 Sep 5;20(9):e0330071. doi: 10.1371/journal.pone.0330071 (PMC12412958; doi:10.1371/journal.pone.0330071)
Supplement: Attachment — Submitted filename: Reply to editor and reviewers.pdf [file pone.0330071.s010.pdf]

Reply to editor

We thank you very much for your time, reading and commenting our manuscript as well for your patience while waiting for our responses.

We are happy to address all the mentioned concerns below and made sure to answer them point by point. To better decipher between the initial comments/questions and our replies, our replies will be written in **RED**. Additionally, when we added direct quotes from the revised manuscript, they are put in "quotation marks" and written in **BLUE** for unchanged parts and **RED** for changed parts.

1. When submitting your revision, we need you to address these additional requirements.

Please ensure that your manuscript meets PLOS ONE's style requirements, including those for file naming. The PLOS ONE style templates can be found at

https://journals.plos.org/plosone/s/file?id=wjVg/PLOSOne_formatting_sample_main_body.pdf and

https://journals.plos.org/plosone/s/file?id=ba62/PLOSOne_formatting_sample_title_authors_affiliations.pdf

Thank you for providing us with the style guidelines. We went through the manuscript and applied everything according to the template.

2. Please note that PLOS ONE has specific guidelines on code sharing for submissions in which author-generated code underpins the findings in the manuscript. In these cases, we expect all author-generated code to be made available without restrictions upon publication of the work. Please review our guidelines at https://journals.plos.org/plosone/s/materials-and-software-sharing#loc-sharing-code and ensure that your code is shared in a way that follows best practice and facilitates reproducibility and reuse.

We agree with you that sharing our code with the community is important. We uploaded the code as a ZIP file to the data repository where all of the relevant data for this work lies.

The full dataset, as well as the p values and the python3 codes can be accessed via the following link:

10.6084/m9.figshare.26862634

3. Thank you for stating in your Funding Statement:

This research was supported by the German Research Foundation (SFB1027) and large-instrument funding under grant number INST 256/542-1 FUGG.

Please provide an amended statement that declares all the funding or sources of support (whether external or internal to your organization) received during this study, as detailed online in our guide for authors at http://journals.plos.org/plosone/s/submit-now. Please also include the statement "There was no additional external funding received for this study." in your updated Funding Statement.

Please include your amended Funding Statement within your cover letter. We will change the online submission form on your behalf.

We changed the Funding Statement according to PLOS ONE's guidelines. Our new Funding statements now is:

"This research was supported by the German Research Foundation (SFB1027) and large-instrument funding under grant number INST 256/542-1 FUGG. The founders had no role in study design, data collection, and analysis, decision to publish, or preparation of the manuscript. There was no additional funding received for this study. "

4. We note that Figures Fig 2G and Figure S3B-C in your submission contain copyrighted images. All PLOS content is published under the Creative Commons Attribution License (CC BY 4.0), which means that the manuscript, images, and Supporting Information files will be freely available online, and any third party is permitted to access, download, copy, distribute, and use these materials in any way, even commercially, with proper attribution. For more information, see our copyright guidelines: http://journals.plos.org/plosone/s/licenses-and-copyright.

The data taken from the mentioned article (Baltes et al., 2022, Frontiers) was also published under CC BY 4.0 licence. For your convenience we added a screenshot of their statement regarding copyright:

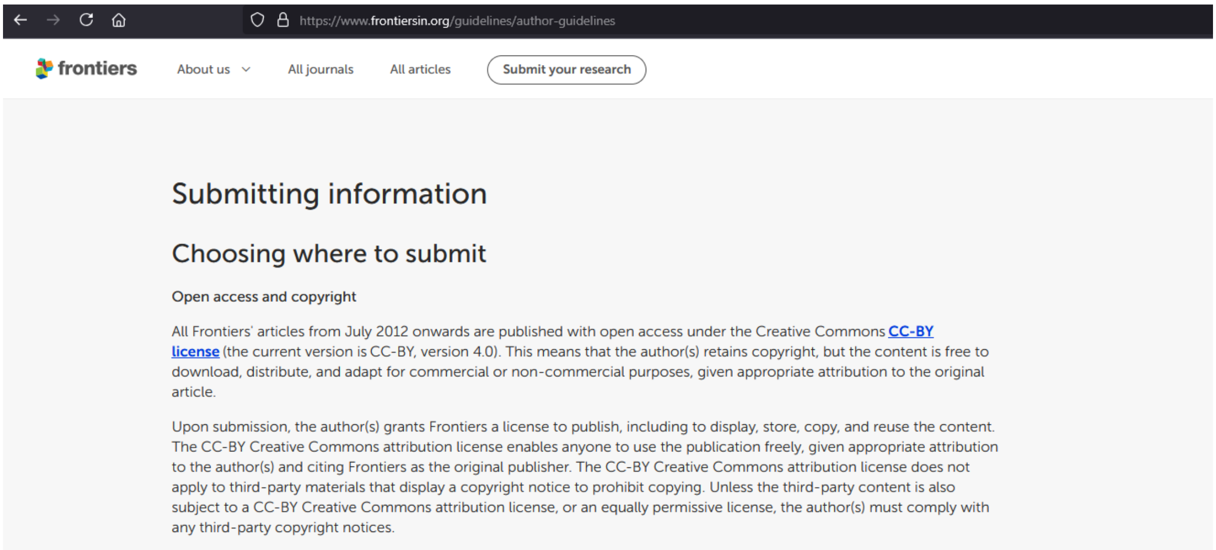

https://www.frontiersin.org/guidelines/author-guidelines

frontiers About us All journals All articles Submit your research

Submitting information

Choosing where to submit

Open access and copyright

All Frontiers' articles from July 2012 onwards are published with open access under the Creative Commons CC-BY license (the current version is CC-BY, version 4.0). This means that the author(s) retains copyright, but the content is free to download, distribute, and adapt for commercial or non-commercial purposes, given appropriate attribution to the original article.

Upon submission, the author(s) grants Frontiers a license to publish, including to display, store, copy, and reuse the content. The CC-BY Creative Commons attribution license enables anyone to use the publication freely, given appropriate attribution to the author(s) and citing Frontiers as the original publisher. The CC-BY Creative Commons attribution license does not apply to third-party materials that display a copyright notice to prohibit copying. Unless the third-party content is also subject to a CC-BY Creative Commons attribution license, or an equally permissive license, the author(s) must comply with any third-party copyright notices.

We require you to either present written permission from the copyright holder to publish these figures specifically under the CC BY 4.0 license, or remove the figures from your submission:

a. You may seek permission from the original copyright holder of Figures Fig 2G and Figure S3B-C to publish the content specifically under the CC BY 4.0 license.

We recommend that you contact the original copyright holder with the Content Permission Form (http://journals.plos.org/plosone/s/file?id=7c09/content-permission-form.pdf) and the following text:

"I request permission for the open-access journal PLOS ONE to publish XXX under the Creative Commons Attribution License (CCAL) CC BY 4.0 (http://creativecommons.org/licenses/by/4.0/). Please be aware that this license allows unrestricted use and distribution, even commercially, by third parties. Please reply and provide explicit written permission to publish XXX under a CC BY license and complete the attached form."

Please upload the completed Content Permission Form or other proof of granted permissions as an "Other" file with your submission.

In the figure caption of the copyrighted figure, please include the following text: "Reprinted from [ref] under a CC BY license, with permission from [name of publisher], original copyright [original copyright year]."

b. If you are unable to obtain permission from the original copyright holder to publish these figures under the CC BY 4.0 license or if the copyright holder's requirements are incompatible with the CC BY 4.0 license, please either i) remove the figure or ii) supply a replacement figure that complies with the CC BY 4.0 license. Please check copyright information on all replacement figures and update the figure caption with source information. If applicable, please specify in the figure caption text when a figure is similar but not identical to the original image and is therefore for illustrative purposes only.

As mentioned above, the data from the cited article (Baltes et al., 2022, Frontiers) was published under CC BY 4.0 license.

5. We notice that your supplementary figures are uploaded with the file type 'Figure'. Please amend the file type to 'Supporting Information'. Please ensure that each Supporting Information file has a legend listed in the manuscript after the references list.

We thank you for pointing this out. For our revised submission, we selected “Supporting Information” for the supplementary figures.

Reviewers' comments:

Reviewer's Responses to Questions

Comments to the Author

1. Is the manuscript technically sound, and do the data support the conclusions?

The manuscript must describe a technically sound piece of scientific research with data that supports the conclusions. Experiments must have been conducted rigorously, with appropriate controls, replication, and sample sizes. The conclusions must be drawn appropriately based on the data presented.

Reviewer #1: Partly

Reviewer #2: Partly

---

2. Has the statistical analysis been performed appropriately and rigorously?

Reviewer #1: Yes

Reviewer #2: Yes

---

3. Have the authors made all data underlying the findings in their manuscript fully available?

The PLOS Data policy requires authors to make all data underlying the findings described in their manuscript fully available without restriction, with rare exception (please refer to the Data Availability Statement in the manuscript PDF file). The data should be provided as part of the manuscript or its supporting information, or deposited to a public repository. For example, in addition to summary statistics, the data points behind means, medians and variance measures should be available. If there are restrictions on publicly sharing data—e.g. participant privacy or use of data from a third party—those must be specified.

Reviewer #1: Yes

Reviewer #2: Yes

---

4. Is the manuscript presented in an intelligible fashion and written in standard English?

PLOS ONE does not copyedit accepted manuscripts, so the language in submitted articles must be clear, correct, and unambiguous. Any typographical or grammatical errors should be corrected at revision, so please note any specific errors here.

Reviewer #1: Yes

Reviewer #2: Yes

---

5. Review Comments to the Author

Please use the space provided to explain your answers to the questions above. You may also include additional comments for the author, including concerns about dual publication, research ethics, or publication ethics. (Please upload your review as an attachment if it exceeds 20,000 characters)

Reviewer #1: The authors have submitted a decently written manuscript describing the effects of a new compound miuraenamide A (MiuA), recently found to affect actin dynamics, on focal adhesions, cell contractility and migration. They show that in cell culture the treatment with MiuA in two cell types reduce average focal adhesion size but increase the number of adhesions. They also find that the strength of these adhesions, despite being higher in number, are less adhesive. The authors also show that amoeboid type migration is unaffected by MiuA treatment. Overall the experiments seem to be well-performed and nearly adequate analysis. However, the overall writing style needs some adjustment for clarity as well as some additional questions need to be addressed before this paper should be accepted for publication in PONE. The following issues should be addressed:

We thank you for pointing out the scientific value of our work. We are glad to address the mentioned concerns and answer them. Our replies will be written in **RED**. Additionally, to highlight changes that took place in the manuscript itself, they were written in **BLUE.**

(1) The way the manuscript is written seems very redundant in many places. For example, the Introduction section is extremely long and is written to include the results. Provide only a quick summary of your results (not going figure by figure) in the introduction and keep the results section that you do have for the detailed explanation of the figures. On the first read of the manuscript, I thought that the introduction's run through of the figures was indeed the results section.

We went through the manuscript and reduced redundancy. We worked on the style in general to improve the readability of the text. Regarding the introduction, we now only provide a short summary of the results in the introduction and simultaneously added more background on MiuA (lines 67-76 and 85-92):

"

Recently, a new compound, miuraenamide A (MiuA), has been used in studies to stabilize actin filaments in living cells and in vitro [16–20]. This new tool allows investigation of the effects of actin stabilization in migration and its underlying mechanisms.

MiuA achieves this stabilizing effect by shifting the conformation of F-actin monomers [16]. MD simulations performed by Wang et al. showed that this change in conformation blocks the binding site of cofilin, an actin depolymerizing factor, which ultimately results in a decreased depolymerization rate of actin filaments and leads to an increased length of actin filaments. This effect has also been verified in vitro by Wang et al. [16]. Additionally, Gegenfurtner et al. tested the transcriptional effects of MiuA [21]. They report a net positive effect on the following categories of genes: Cellular adhesion and spreading, smooth muscle contraction and regulation of lamellipodium assembly. […]

In this new study we showed that MiuA stabilizes and elongates actin filaments in RPE-1 and MEF cells and also affects the number and size of focal adhesions in those cells. We then

measured the adhesion forces of the cells and found that the MiuA treated ones exert a lower adhesion force and energy. Additionally, we placed cells on a patterned soft substrate, which they deform by contracting. Measuring these deformations, we were able to calculate the contractile energy of single cells. We showed that MiuA does not affect the contractile energy of cells. Lastly, we conducted 1D migration experiments with RPE-1 cells and neutrophils confined in PDMS microchannels. MiuA did not affect the amoeboid migration behavior in either cell line.

”

(2) The authors use two cell types for the beginning two figure's worth of experiments but then drop the use of the MEF cells. Why is this? MEF cells are commonly used for cell migration experiments, especially in 2D settings, so some analysis of their behavior upon MiuA treatment would be helpful to the field and further the impact of this paper.

We thank you for pointing this out. We totally agree with you about the interest of analyzing MEF cells with MiuA treatment in detail. In fact, we already did these experiments: the 2D migration experiments in MEF cells are published already in 2022. The data shown in this manuscript are new and complementary to the already published MEF data. However, it now seemed like we dropped the MEF cell line. We clarified this in the main text, however, we understood by your concerns that we need to clarify this even more. We changed the text as follow (lines 77-84):

“

In an earlier study, we demonstrated that MiuA reduces actin filament dynamics, thereby altering the first of the migration processes discussed by Lämmermann et al.: the polymerization rate. Additionally, we observed that MiuA treatment reduced mesenchymal migration in RPE-1 and MEF cells in a 2D unconfined environment [22]. Following our previous work, we wanted to understand the effects of the actin stabilizing compound MiuA on the remaining two migration mechanisms: adhesion and contractility, on amoeboid as well as mesenchymal cell migration. We therefore performed adhesion and contractility measurements on RPE-1 and on MEF cells, and employed amoeboid migration assays in PDMS microchannels using RPE-1 cells and neutrophils.

”

For your convenience, we added the figures in question from the aforementioned article “Actin stabilization in cell migration” (reference [22] in our manuscript) to the following replies. The exact citation is:

Baltes C, Thalla DG, Kazmaier U and Lautenschläger F (2022) Actin stabilization in cell migration. Front. Cell Dev. Biol. 10:931880. doi: 10.3389/fcell.2022.931880

We hope you can appreciate now the overall impact of our work on several cell lines.

(3) On a similar note, the authors don't seem to address 2D migration parameters, which would be greatly beneficial to the broader cell migration community (and again, boost the impact of this publication). I suggest that the authors analyze the effect of MiuA treatment on 2D migration parameters such as speed, persistence, directionality, etc. These parameters are only analyzed in the context of the PDMS confinement channels during amoeboid migration. Is it known how MiuA affects unconfined 2D migration? Such a change in focal adhesions parameters surely affects 2D migration.

As explained in the previous answer, we already performed and analysed 2D migration experiments in RPE-1 and MEF cells. Nonetheless, we now took care to improve our writing to better refer to these data and emphasize the broader picture of our findings, including 2D migration and its respective analysis. In the following we replot for your convenience the subfigure (Fig. 5 E-H) and Si-Figure SI 3 from our previously published article (Baltes et al. 2022):

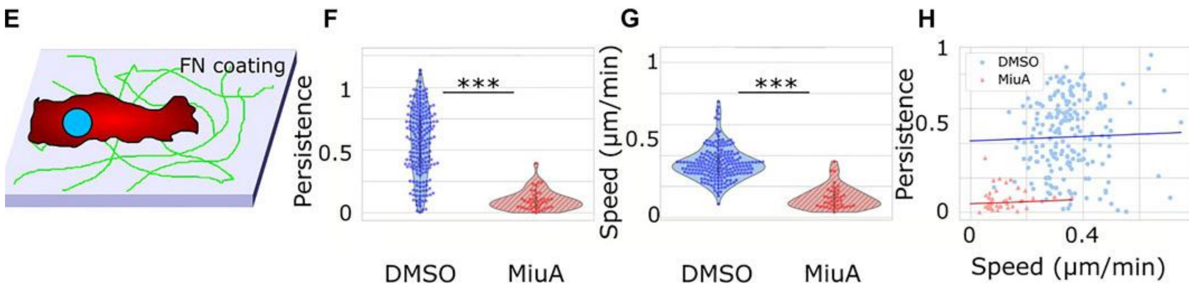

(Figure 5: Migration of RPE-1 cells on fibronectin coated surfaces. (E) Scheme of the experimental setup for 2D migration on a surface. (F), (G)) Violin plot of the persistence and speed of migrating cells. When treated with 20 nM MiuA, the persistence and speed decreased significantly. (H) Plotting the persistence of cells against their speed revealed a slightly positive correlation in both groups. Number of cells 2D: 197 (DMSO), 43 (MiuA).)

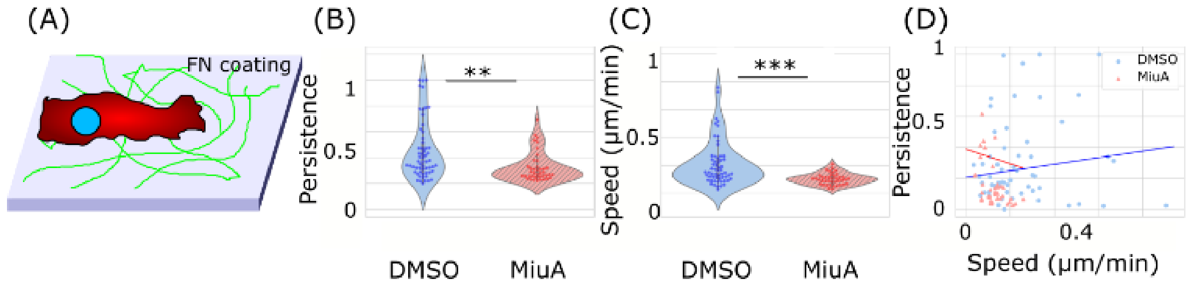

(SI Figure 3 (A) Scheme of the experimental setup for 2D migration on a surface (MEFs). (B), (C) Violin plot of the persistence and speed of migrating MEFs. When treated with 20nM MiuA, the persistence and speed decreased significantly. (D) Plotting the persistence of cells against their speed. Number of MEFs 2D: 58 (DMSO), 40 (MiuA).)

Both figures from the cited article show that MiuA reduces cellular migration in 2D migrating RPE-1 and MEF cells. This aligns well with our observation that 1D migrating RPE-1 cells (without confinement) also reduce their migration parameter after MiuA treatment.

To emphasize the existing, published data, we changed the manuscript as follows (see our reply to question (3)) (lines 77-84):

"

In an earlier study, we demonstrated that MiuA reduces actin filament dynamics, thereby altering the first of the migration processes discussed by Lämmermann et al.: the polymerization rate. Additionally, we observed that MiuA treatment reduced mesenchymal migration in RPE-1 and MEF cells in a 2D unconfined environment [22]. Following our previous work, we wanted to understand the effects of the actin stabilizing compound MiuA on the remaining two migration mechanisms: adhesion and contractility, on amoeboid as well as mesenchymal cell migration. We therefore performed adhesion and contractility measurements on RPE-1 and on MEF cells, and employed amoeboid migration assays in PDMS microchannels using RPE-1 cells and neutrophils.

"

(4) In Figure 1, the authors show only the spreading area and actin filament length analysis for MEF cells but not RPE-1 cells – why? There seems to be a significant change in the spreading area for RPE-1 cells based on the images in panel (1C). Since the authors rely heavily on RPE-1 cells throughout the manuscript, this should not be omitted here.

As before, the analysis of spreading area and actin filament length in RPE-1 cells has already been performed and published in our previous publication. We updated the manuscript to refer to the mentioned publication and explain the differences in the results where RPE-1 cells increase their spreading area with MiuA while MEF cells do not change their spreading area with MiuA. As a reference for you, we include the data on spreading area of RPE-1 cells from our article "actin stabilization in cell migration" here:

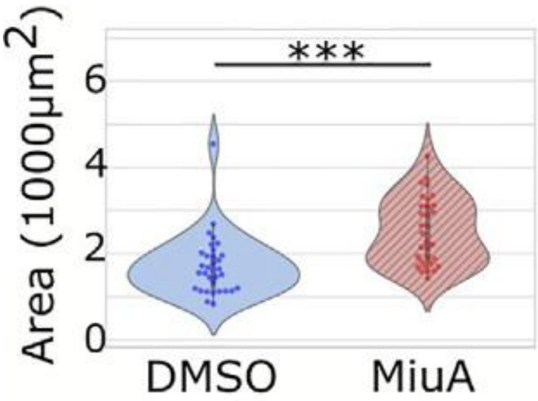

(Figure 4B: Effects of 20 nM MiuA on the spreading area and the number of focal adhesions in RPE-1 cells. (B) The spreading area of MiuA treated cells increased significantly. Number of cells: 35 (DMSO), 34 (MiuA).)

The spreading area in RPE-1 cells increased with MiuA contrary to the results with MEFs. We hypothesize this is caused by the different amounts of MiuA used in the experiments. For RPE-1 cells a concentration of 20nM has been used. However, this concentration proved to be cytotoxic to MEF cells, which is why we reduced the concentration in MEF cells to 5nM. This reduction in concentration might have influenced the effect of MiuA on the spreading area of MEFs.

To emphasize the fact of existing data, we altered the manuscript as follows (lines 231-253):

"

In our previous work [22], we already reported the actin stabilizing effect in RPE-1 cells as well as the reduction of 2D migration in RPE-1 and MEF cells when treated with MiuA. However, we did not look at the effect of MiuA on the actin filament length in MEF. We have therefore now done this by seeding MEF GFP vinculin cells on glass cover slips coated with fibronectin (25 µg/ml) and allowing them to fully adhere. Fig. 1 (a) shows an example of fully adhered spread cells in a side-by-side comparison between RPE-1 and MEF cells. We found that the MEF cells treated with MiuA did not increase their spreading area (Fig. 1 (b)), remaining at around 550 μm^2, while the average length of their actin filaments increased from 10.2 µm in untreated cells to 19.1 µm in MiuA treated cells (Fig 1 (c)). This effect partially aligns with the effects in RPE-1 cells that we already reported [22]. In both cell lines the average length of actin filaments increased but only RPE-1 cells showed an additional increase in spreading area. We expected to see an increase in the spreading area of MEF cells as well, since Gegenfurtner et al. [21] reported that MiuA positively influences genes related to cell spreading. We hypothesize that this effect in our case only takes place in RPE-1 cells and not in MEF cells since the concentration of MiuA was higher in RPE-1 cells (20 nM) compared to MEF cells (5 nM). When we tested different concentrations, we found 20 nM to be cytotoxic for MEF cells and therefore reduced to concentration of MiuA accordingly. Additionally, when we measured the aspect ratio and circularity of RPE-1 and MEF cells we found no significant differences (Fig. 1 (d), (e), (g)) or a low significance (Fig. 1 (f), p-value ~ 0.04). The results align with our expectations. We found RPE-1 cells increased their spreading area but initially exhibited a uniform distribution of actin filaments. As all cells were treated post-adherence, we did not anticipate MiuA to alter this distribution or cell geometry. Conversely, we found MEF cells did not increase their spreading area. We propose that in MEF cells MiuA only stabilized pre-existing actin filaments and elongated within the cell's established borders, leaving the cell geometry unaffected. However, we conclude that the treatment with MiuA leads to an elongation and stabilization of actin filaments within living cells regardless of the cell line.

"

Reviewer #2: The authors demonstrate the effect of an actin-stabilizing agent (MiuA) on the adhesivity, contractility and migration of two cell types. They show that although focal adhesions are impacted by this drug, the contractility and ability to migrate in the amoeboid mode are not.

These findings are an interesting addition to a growing body of work on cell mechanics and cell-substrate mechanical interactions. However, some more mechanistic insights and explanation of the data could strengthen this study, and some of the rationale is not well-described. Some specific concerns and questions are:

We thank you for pointing out the importance of our work and the possible contribution to the field of cell mechanics and cell-substrate mechanical interactions. We are glad to address all concerns and reply to the questions below. Our replies are written in **RED.** Additionally, to highlight changes that took place in the manuscript itself, they were written in **BLUE.**

1. The Introduction paragraphs 3 and 4 are essentially a summary of the Results, which is unconventional. As a reader, I am interested in knowing more about the rationale and background of the study.

We shortened the "result" part of the introduction and focused more on the background. In the following we answer all of your concerns point by point and will give the updated text.

what is known about the biochemical mechanism of how MiuA works: does it inhibit actin polymerization or severing, for example?

MiuA does interact with F-actin monomers. The current model of its interaction (provided by Gegenfurtner et al. 2022 via MD simulations) tells us that MiuA shifts the conformation in F-actin in a way that the binding side of cofilin is blocked. Cofilin is an actin depolymerizing factor, which means that its inhibition ultimately leads to elongated and stabilized actin filaments. This effect of actin elongation and stabilization has experimentally been verified in vitro by Gegenfurtner et al. 2022.

We added this to the text as follows (lines 67-74):

"

Recently, a new compound, miuraenamide A (MiuA), has been used in studies to stabilize actin filaments in living cells and in vitro [16–20]. This new tool allows investigation of the effects of actin stabilization in migration and its underlying mechanisms.

MiuA achieves this stabilizing effect by shifting the conformation of F-actin monomers [16]. MD simulations performed by Wang et al. showed that this change in conformation blocks the binding site of cofilin, an actin depolymerizing factor, which ultimately results in a decreased

depolymerization rate of actin filaments and leads to an increased length of actin filaments. This effect has also been verified in vitro by Wang et al. [16].

"

how does it interact with actin binding proteins?

Apart from the aforementioned indirect interaction with cofilin, there is no literature in the field. We agree with you that this is an interesting topic. However, studying this would be out of scope for our manuscript.

what are the pros and cons of using this as a stabilizng agent vs. jusplakinolide or phalloidin?

Although jasplakinolide and phalloidin are also known for stabilizing actin filaments, we chose MiuA as there are disadvantages of using either of the two. Phalloidin cannot enter cells without membrane permeabilization (except for specific liver cells), therefore limiting its usage in our experiments in living cells. Jasplakinolide can enter living cells, but literature tells us that its effect is highly dosage dependent, where a high concentration of jasplakinolide might induce actin depolymerization instead of actin stabilization. Additionally, there are reports that jasplakinolide might induce a delayed cell death via cytotoxicity.

This was stated in the manuscript starting at line 46. However, we admit that the description in the manuscript was too short, which is why we extended it further (lines 54-74):

"

Substances that depolymerize the actin network, such as cytochalasin D or latrunculin A, have been used to reliably study the effects of a disassembled actin network on cellular functions, including migration [4–10]. Nevertheless, the stabilization of actin filaments has proven to be a significant challenge. The two most prominent actin stabilizing agents are phalloidin and jasplakinolide. The first is unable to pass through the cell membrane [11], while the effectiveness of the second is dependent on the concentration used and the duration of exposure, with optimal results requiring precise calibration [12].

Nevertheless, the actin stabilization compound jasplakinolide has been used in various studies. It has been shown to reduce migration in MDA-MB-231 and MCF7 cells in a dose dependent manner [10,13] but shows the contrary effect on A549 cells [10]. Additionally, experiments on cellular growth and apoptosis showed that jasplakinolide not only reduces cellular growth but is also capable of inducing apoptosis [14,15].

The versatile use of jasplakinolide reflects the interest in the community of the interplay between actin stabilization and cellular mechanics. However, jasplakinolide remained the only available actin stabilizer suitable for live cell experiments for a long time.

Recently, a new compound, miuraenamide A (MiuA), has been used in studies to stabilize actin filaments in living cells and in vitro [16–20]. This new tool allows investigation of the effects of actin stabilization in migration and its underlying mechanisms.

MiuA achieves this stabilizing effect by shifting the conformation of F-actin monomers [16]. MD simulations performed by Wang et al. showed that this change in conformation blocks the binding site of cofilin, an actin depolymerizing factor, which ultimately results in a decreased depolymerization rate of actin filaments and leads to an increased length of actin filaments. This effect has also been verified in vitro by Wang et al. [16].

"

what are the RPE1 and MEF cell types and why are they the suitable candidates in this study?

We agree with you that a clearer depiction of the used cell lines would benefit the manuscript. For this we added information about the cell lines as well as our reasoning why we used them to the manuscript (lines 35-46):

"

In general cells show two distinct modes of migration. They can either migrate in an adhesion-based manner, so called mesenchymal migration, or rely on friction forces and contractility to propel themselves forward, so called amoeboid migration. These two modes are not exclusive as some cells can switch between them and even display modes of migration that have characteristics from both [1]. For our research we decided to work with three different cell lines: RPE-1 (Retinal Pigment Epithelial) cells, MEF (Mouse Embryonic Fibroblasts) cells, and neutrophils. RPE-1 cells are a commonly used cell line that is capable of adhesion and migration. However, we specifically chose RPE-1 cells for their ability to switch between mesenchymal and amoeboid migration [1]. In a similar way, MEF cells are an adherent cell line and perform predominantly mesenchymal migration, therefore allowing us to compare them to RPE-1 cells. Lastly, neutrophils are non-adherent cells, which are differentiated from HL60 cells. They are used as a model for amoeboid migration [2] and serve our research as a control system to validate our findings in amoeboid migration of RPE-1 cells.

"

2. In Fig. 1, it is abrupt to start with quantification before showing cell images first. Also, why quantification is shown only for MEF cell type only in Figs 1a and 1b and not for RPE1?

You are right, you convinced us that it is more intuitive to first show qualitative images before we quantify our data. Please find below our new Figure 1, where the first panel (a) now depicts qualitative images of RPE-1 and MEF cells with and without MiuA. We also added new panels (d)-(g), as you requested in question 4., to analyse the aspect ratios and circularity of the cells. For both we either find no significance or only a weak one ($p \sim 0.04$). This aligns with our expectations, as we hypothesized that the elongation and stabilization of actin shows no preferred direction that would ultimately lead to a change in geometry. We updated the text accordingly (lines 245-249):

"

Additionally, when we measured the aspect ratio and circularity of RPE-1 and MEF cells we found no significant differences (Fig. 1 (d), (e), (g)) or a low significance (Fig. 1 (f), p-value ~ 0.04). The results align with our expectations. We found RPE-1 cells increased their spreading area but initially exhibited a uniform distribution of actin filaments. As all cells were treated post-adherence, we did not anticipate MiuA to alter this distribution or cell geometry.

"

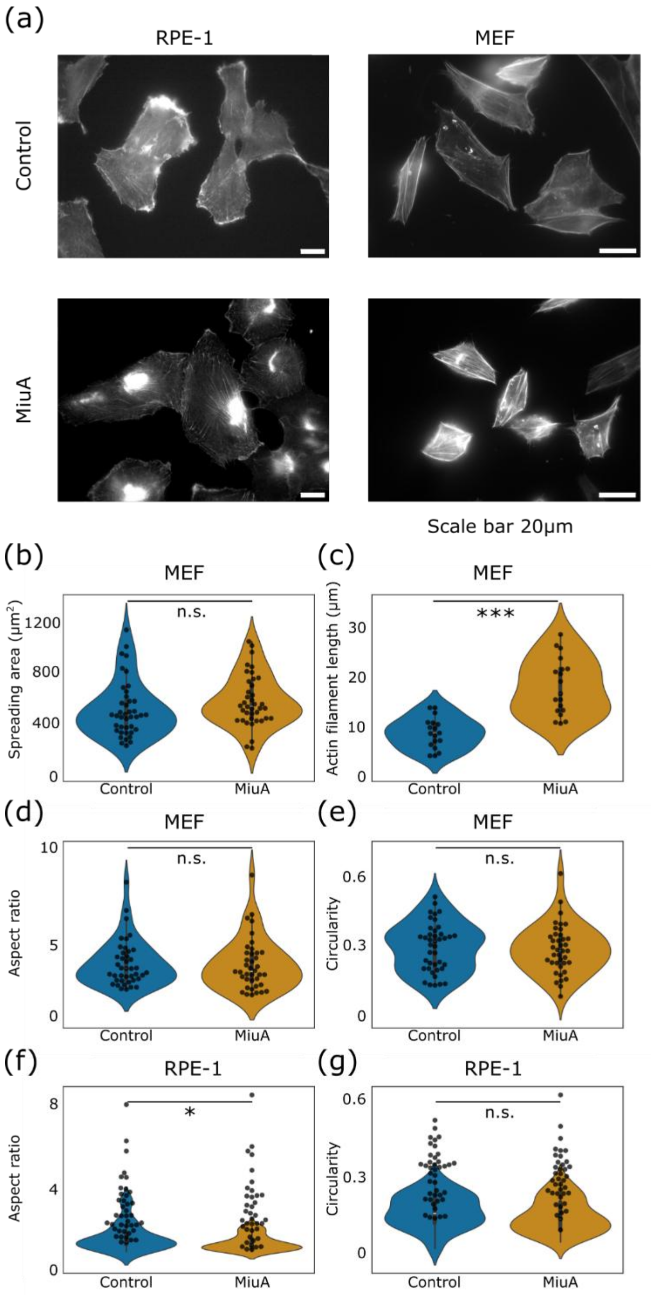

(Fig 1. Effect of MiuA on actin. (a) Representative images of the actin cytoskeleton in RPE-1 LifeAct mCherry and MEF GFP vinculin cells when treated with MiuA. Top: control cells. Bottom: MiuA treated cells (20 nM for RPE-1 LifeAct mCherry and 5 nM for MEF GFP vinculin). Actin filaments in MEF GFP vinculin cells were stained with Phalloidin-iFluor 555. Scale bar 20 μm. (b) Spreading area of MEF GFP vinculin cells seeded on a fibronectin-coated glass surface; untreated (control) cells and cells treated with 5 nM MiuA. (c) Mean length of actin filaments

*in MEF GFP vinculin cells, spread on fibronectin-coated glass surface. Effect of MiuA on the aspect Ratio of MEF (d) and RPE-1 (f) cells. Effects of MiuA on the circularity of MEF (e) and RPE-1 (g) cells. Number of cells: MEF: Control: 41, MiuA: 38; RPE-1: Control: 38, MiuA: 35. A statistical analysis was performed using Student's t test. n.s.: p>0.05; *: p<0.05; **: p<0.01; and ***: p<0.005.)*

Concerning the quantification on RPE1 cells: The data on RPE-1 actin filament length and spreading area has already been published by us in 2022 (doi: 10.3389/fcell.2022.931880). To reduce redundancy, we only refer to this already published data instead of replotting it again. However, we now went through the manuscript and added emphasis on these already published data. As a reference for you, we show the figure from the previous article here:

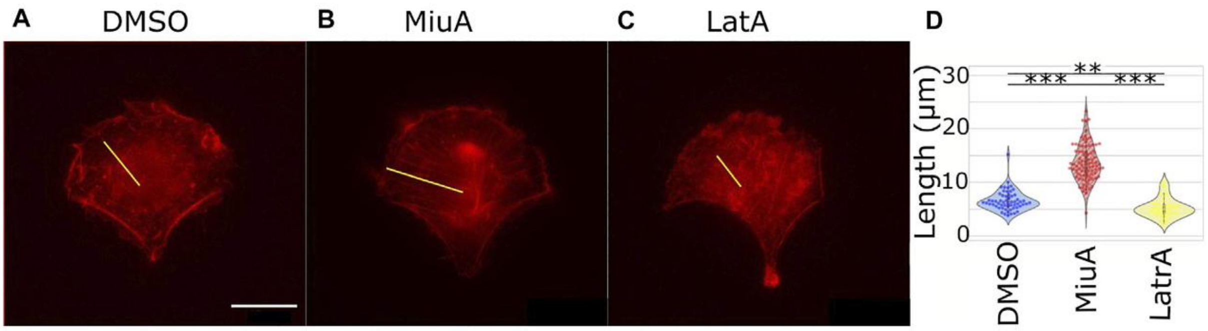

(FIGURE 3. Length of actin filaments in geometrically identical RPE-1 lifeAct mCherry cells placed on a crossbow pattern. (A) Control cells (DMSO). (B) MiuA treated cells. (C) Latrunculin A treated cells. A yellow line indicates examples of how the length of actin filaments was measured. (D) Violinplots of the mean length of actin filaments in cells. Treatment with MiuA increases actin filament length, the treatment with Latrunculin A decreases filament length. Number of cells: 58 (DMSO), 88 (MiuA), 38 (Latrunculin A). Scale bar is 15 µm.)

The elongation of actin filaments is present in all our experiments, as our new manuscript shows the same results but in MEFs.

Regarding the spreading area: These data have also been published in the mentioned article (Baltes et al., 2022, Frontiers). Here we replot the data for your convenience:

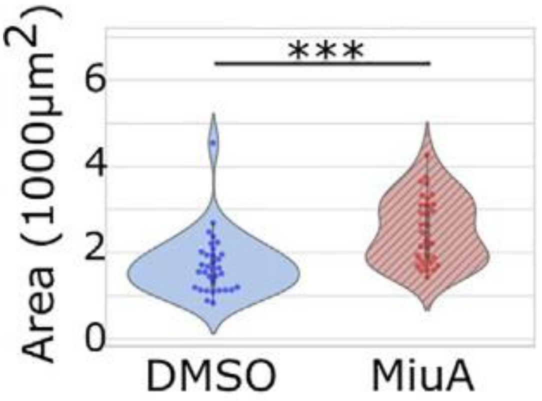

(Figure 4B: Effects of 20 nM MiuA on the spreading area and the number of focal adhesions in RPE-1 cells. (B) The spreading area of MiuA treated cells increased significantly. Number of cells: 35 (DMSO), 34 (MiuA).)

The spreading area in RPE-1 cells increased with MiuA contrary to the results with MEFs. We hypothesize this is caused by the different amounts of MiuA used in the experiments. For RPE-1 cells a concentration of 20nM has been used. However, this concentration proved to be cytotoxic to MEF cells, which is why we reduced the concentration in MEF cells to 5nM. This reduction in concentration might have influenced the effect of MiuA on the spreading area of MEFs.

To clarify and address the addressed concerns, we changed our manuscript as follows (lines 231-245):

"

In our previous work [22], we already reported the actin stabilizing effect in RPE-1 cells as well as the reduction of 2D migration in RPE-1 and MEF cells when treated with MiuA. However, we did not look at the effect of MiuA on the actin filament length in MEF. We have therefore now done this by seeding MEF GFP vinculin cells on glass cover slips coated with fibronectin (25 µg/ml) and allowing them to fully adhere. Fig. 1 (a) shows an example of fully adhered spread cells in a side-by-side comparison between RPE-1 and MEF cells. We found that the MEF cells treated with MiuA did not increase their spreading area (Fig. 1 (b)), remaining at around 550 µm^2, while the average length of their actin filaments increased from 10.2 µm in untreated cells to 19.1 µm in MiuA treated cells (Fig 1 (c)). This effect partially aligns with the effects in RPE-1 cells that we already reported [22]. In both cell lines the average length of actin filaments increased but only RPE-1 cells showed an additional increase in spreading area. We expected to see an increase in the spreading area of MEF cells as well, since Gegenfurtner et al. [21] reported that MiuA positively influences genes related to cell spreading. We hypothesize that this effect in our case only takes place in RPE-1 cells and not in MEF cells since the concentration of MiuA was higher in RPE-1 cells (20 nM) compared to MEF cells (5 nM). When we tested different concentrations, we found 20 nM to be cytotoxic for MEF cells and therefore reduced to concentration of MiuA accordingly.

"

3. Fig. 1c focuses on individual cells, but to be convincing the authors should also show a wider field of view with many cells, so that the reader can see the representative behavior.

Since all of our quantification was performed in single cells, we had illustrated this by only showing one cell. However, we are happy to exchange the original images with new ones showing more than one cell. Although we already presented the new figure one in the reply to question 2., we insert the subfigure showing only the mentioned panel here again:

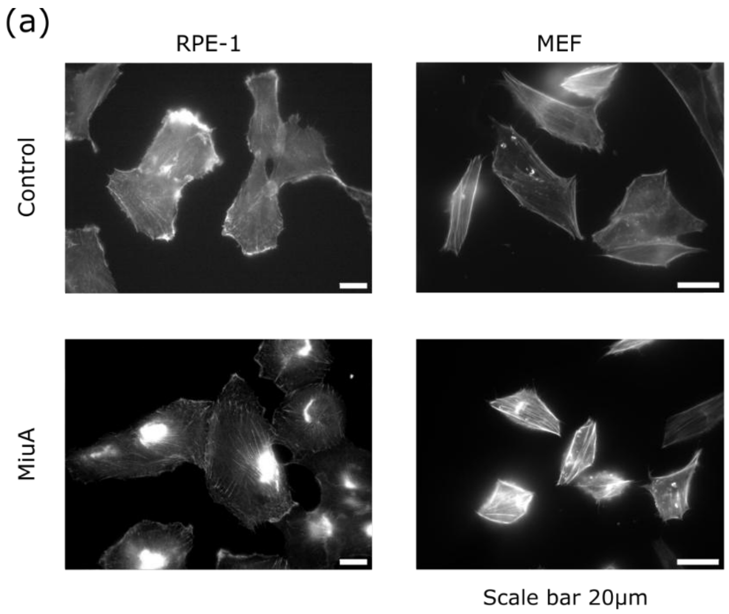

(Fig 1. Effect of MiuA on actin. (a) Representative images of the actin cytoskeleton in RPE-1 LifeAct mCherry and MEF GFP vinculin cells when treated with MiuA. Top: control cells. Bottom: MiuA treated cells (20 nM for RPE-1 LifeAct mCherry and 5 nM for MEF GFP vinculin). Actin filaments in MEF GFP vinculin cells were stained with Phalloidin-iFluor 555. Scale bar 20 µm.)

4. The MiuA treated MEF cell shown has an interesting spindle-like morphology. Have the authros tried to quantify this effect? A quantification of the cell aspect ratio would reflect this, for example. It will be interesting to see how cell morphology is affected in addition to cell area, by MiuA.

We want to thank you for pointing this out. Initially we did not analyse this effect, but we now did. We added the analysis of the circularity and aspect ratio of RPE-1 and MEF cells to our manuscript and dedicated a new panel (d)-(g) in Figure 1 to this quantification. However, we already addressed this in question 2. To avoid doubling our answers, we would like to refer to our reply to question 2.

5. There should be some explanation, at least qualitative, why the contractility is not affected although adhesivity is.

We agree with you and added our hypothesis on why contractility is not affected although adhesion is to the text. We added the following phrases to the manuscript (lines 350-356):

"

Although MiuA reportedly changes the architecture of the actin network in cells, which has an impact on cellular mechanics such as adhesion, we do not know of any reports about the effects of MiuA on the interaction between actin and myosin motor proteins. Since MiuA does not disrupt the actin network and presumably does not block myosin from actin filaments, we

assume that the interaction between the two is unchanged in our experiments. This explains why we observe no changes in contractility in our experiments. However, further studies on the interactions between actin, MiuA and motor proteins, especially on co-localization are required to fully answer this question.

"

6. Why are neutrophils intriduced for motility measurements, and not MEF? do these not migrate?? there could be a cartoon to illustrate how persistence is measured. In addition to adhesions, can the 1D vs 2D nature of the migration also play a role?

We used neutrophils as they are seen as a model system for amoeboid migration. As part of the immune system they tend towards a more amoeboid way of migration. On the other hand, RPE-1 one cells can switch between different modes of migration. The usage of neutrophils allowed us to test whether the observed effects of MiuA on amoeboid migration depend on the used cell type, which we can falsify with our migration data on RPE-1 and neutrophils. For clarification we added the following text to our introduction (lines 39-46):

"

For our research we decided to work with three different cell lines: RPE-1 (Retinal Pigment Epithelial) cells, MEF (Mouse Embryonic Fibroblasts) cells, and neutrophils. The first are a commonly used cell line that is capable of adhesion and migration. However, we specifically chose them for their ability to switch between mesenchymal and amoeboid migration [1]. In a similar way, MEF cells are an adherent cell line and perform predominantly mesenchymal migration, therefore allowing us to compare them to RPE-1 cells. Lastly, neutrophils are non-adherent cells, which are differentiated from HL60 cells. They are used as a model for amoeboid migration [2] and serve our research as a control system to validate our findings in amoeboid migration of RPE-1 cells.

"

Regarding the migration of MEF cells: we already tested the effect of MiuA on 2D MEF migration and published these findings in 2022 (doi: 10.3389/fcell.2022.931880). Here, we also want to refer to our earlier published paper, showing the effect of MiuA on the migration of MEFs and RPE-1 cells on a 2D fibronectin coated surface with the following to figures:

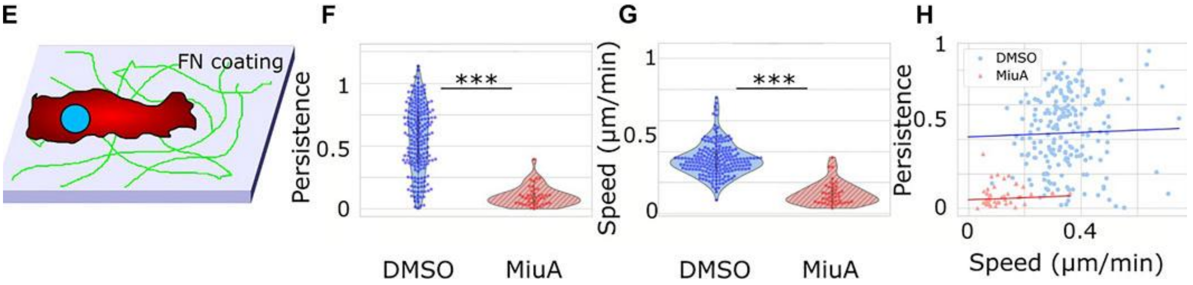

(Figure 5: Migration of RPE-1 cells on fibronectin coated surfaces. (E) Scheme of the experimental setup for 2D migration on a surface. (F), (G)) Violin plot of the persistence and speed of migrating cells. When treated with 20 nM MiuA, the persistence and speed decreased significantly. (H) Plotting the persistence of cells against their speed revealed a slightly positive correlation in both groups. Number of cells 2D: 197 (DMSO), 43 (MiuA).)

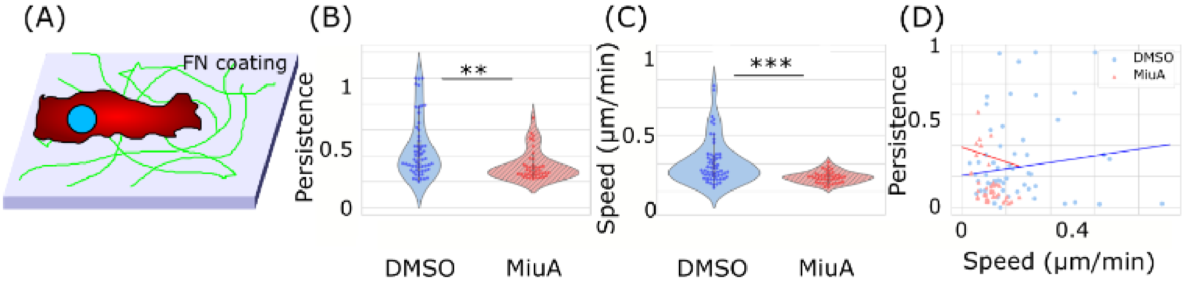

(SI Figure 3 (A) Scheme of the experimental setup for 2D migration on a surface (MEFs). (B), (C) Violin plot of the persistence and speed of migrating MEFs. When treated with 20nM MiuA, the persistence and speed decreased significantly. (D) Plotting the persistence of cells against their speed. Number of MEFs 2D: 58 (DMSO), 40 (MiuA).)

Both figures from the article show that MiuA reduces cellular migration in 2D migrating RPE-1 cells and MEFs. Which aligns well with the observations that 1D migrating RPE-1 cells do as well reduce their migration after MiuA treatment. We added this to the manuscript as follows (line 231-233):

"

In our previous work [22], we already reported the actin stabilizing effect in RPE-1 cells as well as the reduction of 2D migration in RPE-1 and MEF cells when treated with MiuA. However, we did not look at the effect of MiuA on the actin filament length in MEF.

"

We also agree with you, that a sketch depicting our understanding of persistence might facilitate the reading of the figure as a whole. For this we added a new panel (a) to Figure 5.

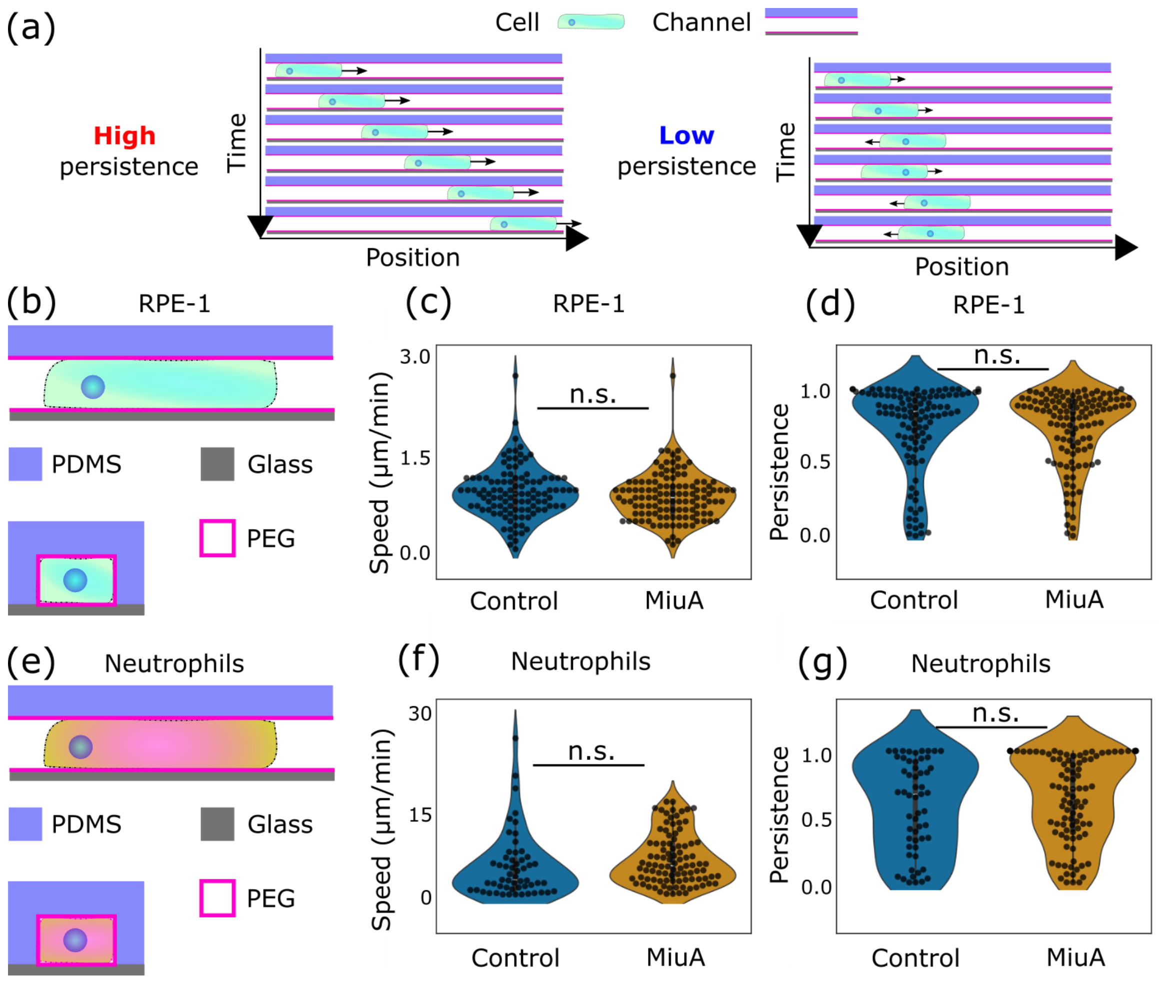

*(**Fig 5. Effects of MiuA on migrating cells**. (a) Visual representation of "persistence". A high persistence means little directional changes, whereas a low persistence indicates a cell that changes its direction a lot. Schematic representation of amoeboid migrating cells (b, e). Under confinement, RPE-1 LifeAct mCherry cells show no differences in speed and persistence (c, d). The same results occur when neutrophils migrate in confinement (f, g). A statistical analysis was performed using Student's t test. n.s.: p>0.05; *: p<0.05; **: p<0.01; and ***: p<0.005. Number of cells: RPE-1: 109 control and 119 MiuA. Neutrophils: 56 control and 102 MiuA.)*

Additionally, we added the following phrases to the manuscript (line 383-385):

"

By "persistence" in this study we refer to the tendency of cells to change their direction while migrating. The value of this persistence lies between 0 (constantly changing direction) and 1 (no changes in direction).

"

7. Similar to Introduction, the Conclusion could be stronger if there was some discussion of the broader context of this work. How does jusplakinolide affect these quantities: cell adhesivity, contractility, and migration for example?

We thank the reviewer for pointing this out. We now added more text about jasplakinolide and its effects on cellular migration (lines 54-66):

"

Substances that depolymerize the actin network, such as cytochalasin D or latrunculin A, have been used to reliably study the effects of a disassembled actin network on cellular functions, including migration [4–10]. Nevertheless, the stabilization of actin filaments has proven to be a significant challenge. The two most prominent actin stabilizing agents are phalloidin and jasplakinolide. The first is unable to pass through the cell membrane [11], while the effectiveness of the second is dependent on the concentration used and the duration of exposure, with optimal results requiring precise calibration [12].

Nevertheless, the actin stabilization compound jasplakinolide has been used in various studies. It has been shown to reduce migration in MDA-MB-231 and MCF7 cells in a dose dependent manner [10,13] but shows the contrary effect on A549 cells [10]. Additionally, experiments on cellular growth and apoptosis showed that jasplakinolide not only reduces cellular growth but is also capable of inducing apoptosis [14,15].

The versatile use of jasplakinolide reflects the interest in the community of the interplay between actin stabilization and cellular mechanics. However, it remained the only available actin stabilizer suitable for live cell experiments for a long time.

"

8. The images provided are low resolution at least in the reviewer's copy. Perhaps the images analyzed should also be shared in high resolution alongside the supplementary data.

We are sorry to hear that the resolution is low in the provided pictures. This time, we uploaded the figures in a higher resolution as in the initial submission.

9. There is some informal/colloquial use of English in some places that seem out of place in a technical document. Sentences should not begin with "And" as in line 63 or 88, and "very well" is not a quantifiable statement in line 76, just to take two examples. The authors could use better human proofreading in addition to using AI-based tools which are not often adequate for this purpose.

We want to thank the reviewer for pointing this out. We read the manuscript thoroughly and corrected any mistakes as well as evaded informal language.

6. PLOS authors have the option to publish the peer review history of their article (what does this mean?). If published, this will include your full peer review and any attached files.

If you choose "no", your identity will remain anonymous but your review may still be made public.

Do you want your identity to be public for this peer review? For information about this choice, including consent withdrawal, please see our Privacy Policy.

Reviewer #1: No

Reviewer #2: No
