# Supplementary material for: Modulation of cellular adhesion, contractility, and migration by MiuA: A comprehensive analysis of its biomechanical impact
Source: PLoS One. 2025 Sep 5;20(9):e0330071. doi: 10.1371/journal.pone.0330071 (PMC12412958; doi:10.1371/journal.pone.0330071)
Supplement: Attachment — Submitted filename: Reply to Editor and reviewer.pdf [file pone.0330071.s011.pdf]

Reply to Editor

We thank you and the referees for your time and helpful comments on our manuscript.

Below we address all the mentioned concerns point by point. To better decipher between the initial comments/questions and our replies, we show our replies in **RED**.

Reviewers' comments:

Reviewer's Responses to Questions

Comments to the Author

1. If the authors have adequately addressed your comments raised in a previous round of review and you feel that this manuscript is now acceptable for publication, you may indicate that here to bypass the "Comments to the Author" section, enter your conflict of interest statement in the "Confidential to Editor" section, and submit your "Accept" recommendation.

Reviewer #1: All comments have been addressed

Reviewer #2: (No Response)

2. Is the manuscript technically sound, and do the data support the conclusions?

The manuscript must describe a technically sound piece of scientific research with data that supports the conclusions. Experiments must have been conducted rigorously, with appropriate controls, replication, and sample sizes. The conclusions must be drawn appropriately based on the data presented.

Reviewer #1: Yes

Reviewer #2: Yes

3. Has the statistical analysis been performed appropriately and rigorously?

Reviewer #1: Yes

Reviewer #2: Yes

4. Have the authors made all data underlying the findings in their manuscript fully available?

The PLOS Data policy requires authors to make all data underlying the findings described in their manuscript fully available without restriction, with rare exception (please refer to the Data Availability Statement in the manuscript PDF file). The data should be provided as part of the manuscript or its supporting information, or deposited to a public repository. For example, in addition to summary statistics, the data points behind means, medians and variance measures should be available. If there are restrictions on publicly sharing data—e.g. participant privacy or use of data from a third party—those must be specified.

Reviewer #1: Yes

Reviewer #2: Yes

5. Is the manuscript presented in an intelligible fashion and written in standard English?

PLOS ONE does not copyedit accepted manuscripts, so the language in submitted articles must be clear, correct, and unambiguous. Any typographical or grammatical errors should be corrected at revision, so please note any specific errors here.

Reviewer #1: Yes

Reviewer #2: Yes

6. Review Comments to the Author

Please use the space provided to explain your answers to the questions above. You may also include additional comments for the author, including concerns about dual publication, research ethics, or publication ethics. (Please upload your review as an attachment if it exceeds 20,000 characters)

Reviewer #1:

The authors have satisfied all of my concerns. I recommend an acceptance of this manuscript for publication.

We thank the reviewer for their positive evaluation and recommendation. We appreciate the constructive feedback throughout the review process.

Reviewer #2:

The authors have significantly improved the clarity of the manuscript in this revised version. They have now answered most of my questions, but I have some more remaining comments which I believe should be addressed before final acceptance.

We sincerely thank the reviewer for their continued engagement and thoughtful feedback. We appreciate the positive assessment of the manuscript's improved clarity and address the remaining comments in detail below.

1.The authors explain that the some of the quantification for RPE-1 cells, corresponding to the results in Fig. 1, were carried out in their previous publication, Ref.22. However, in that study, the MiuA-treated RPE-1 cells increased their spread area, whereas MiuA-treated MEF cells showed no change in spread area. They think this puzzling result is because different doses of MiuA were used for the two cell lines. This raises the obvious question: why not do the same dosage for both cell lines to establish this? Is there any practical limitation to this?

We thank the reviewer for this important question. Initially, we conducted all the experiments with 20nM MiuA. However, during the experiments with MEF cells, we observed that this concentration led to increased cell death, indicating toxicity in this cell type. To avoid confounding effects due to reduced viability, we opted to use a lower concentration of MiuA for MEF cells.

We agree that a systematic dose–response analysis is necessary to directly compare the effects of MiuA across different cell types. In response to this comment, we have now included additional experiments in which we treat RPE-1 WT cells with 5 nM MiuA. We quantified both their spreading area and actin filament length. These new results are presented in the revised manuscript and summarized in the newly added Supplementary Figure S2.

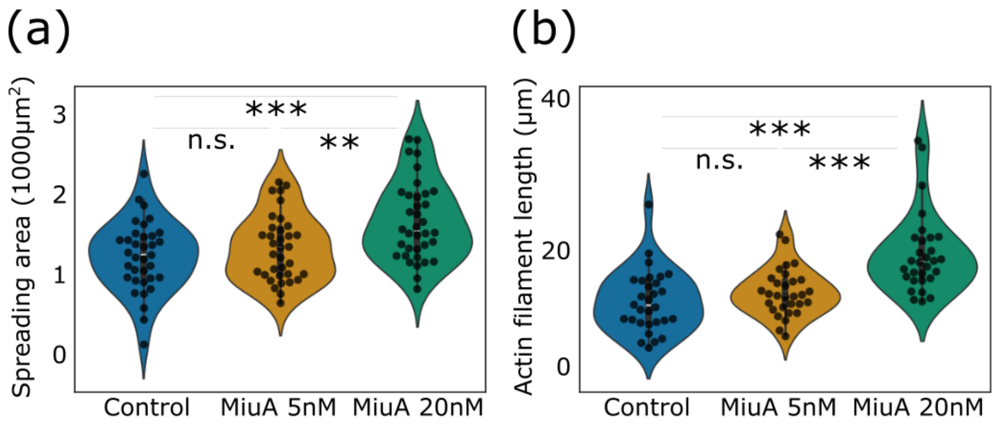

Fig S2. Dosage dependent effect of MiuA on RPE1 cells. (a) Spreading area of RPE-1 wt cells on a fibronectin (25μg/ml) coated glass surface. The treatment with 20nM of MiuA significantly increased the spreading area, whereas 5nM showed no significant effect. (b) Mean actin filament length per cell in RPE-1 wt cells. Treatment with 20nM MiuA significantly increased the mean actin

*filament length, while 5nM showed no significant effect. Number of cells: 35 (a), 30 (b). A statistical analysis was performed using Student's t test. n.s.: p>0.05; *: p<0.05; **: p<0.01; and ***: p<0.005.*

2. Related to above, the authors discuss that one of the disadvantages of using jasplakinolide is that its effect is highly dosage dependent. However, they also found that MiuA's effect is also dosage dependent. How sensitive is this dosage dependence? Have they tried for example, different doses of MiuA on RPE-1 or MEF?

We thank the reviewer for this insightful comment and the opportunity to clarify an important distinction. While both jasplakinolide and MiuA show dose-dependent effects, they do so in qualitatively different ways. Jasplakinolide has been reported to exert opposing effects depending on its concentration—acting as both an actin stabilizer and, paradoxically, an actin destabilizer (Bubb et al., 2000). We now clarify this point in the revised manuscript (lines 62–64), including an updated reference to Bubb et al. (now Ref. [16]):

"Additionally, experiments on cellular growth and apoptosis showed that jasplakinolide not only reduces cellular growth but is also capable of inducing apoptosis [14,15], as well as actin depolymerization [16]."

In contrast, we have not observed such qualitative reversals with MiuA. Its effects appear more straightforwardly dose-dependent—higher concentrations enhance actin filament stability and cell spreading, but toxicity becomes a limiting factor. Indeed, when we applied 20 nM MiuA to MEF cells (the concentration used for RPE-1 cells), we observed increased cell death, prompting us to lower the dose to 5 nM for MEF experiments.

Following your suggestion, we also treated RPE-1 cells with 5 nM MiuA. As shown in the newly added Supplementary Figure S2, this led to a moderate increase in actin filament length and spreading area, although the effect was not statistically significant. These results suggest that MiuA's activity can be fine-tuned in a dose-dependent manner, without evidence of opposing effects as seen with jasplakinolide. This clarification has been added to the manuscript (line 254):

"Treating RPE-1 cells with a lower concentration of MiuA (5 nM) resulted in longer actin filaments and an increased spreading area, but not significantly (Fig. S2)."

3. In Fig. 1a, the MiuA treated MEF cells shown look distinctly smaller by eye than the control MEF cells. Why is this? Is this image not a representative sample of the whole population of cells? In this case, it might be more convincing to choose a different image, or show additional images in the SI.

We thank the reviewer for this observation. The image shown in Fig. 1a was selected because it offers a clear visualization of actin filaments with minimal background noise. However, we acknowledge that the depicted MiuA-treated MEF cell appears smaller, which may raise concerns about representativeness.

In response, we have included a new Supplementary Figure (Fig. S1) presenting five additional examples of MEF cells, both untreated and treated with MiuA. These images illustrate that the apparent size difference in Fig. 1a is not representative of the overall population. We hope this addition addresses the concern and provides a more comprehensive view of the data.

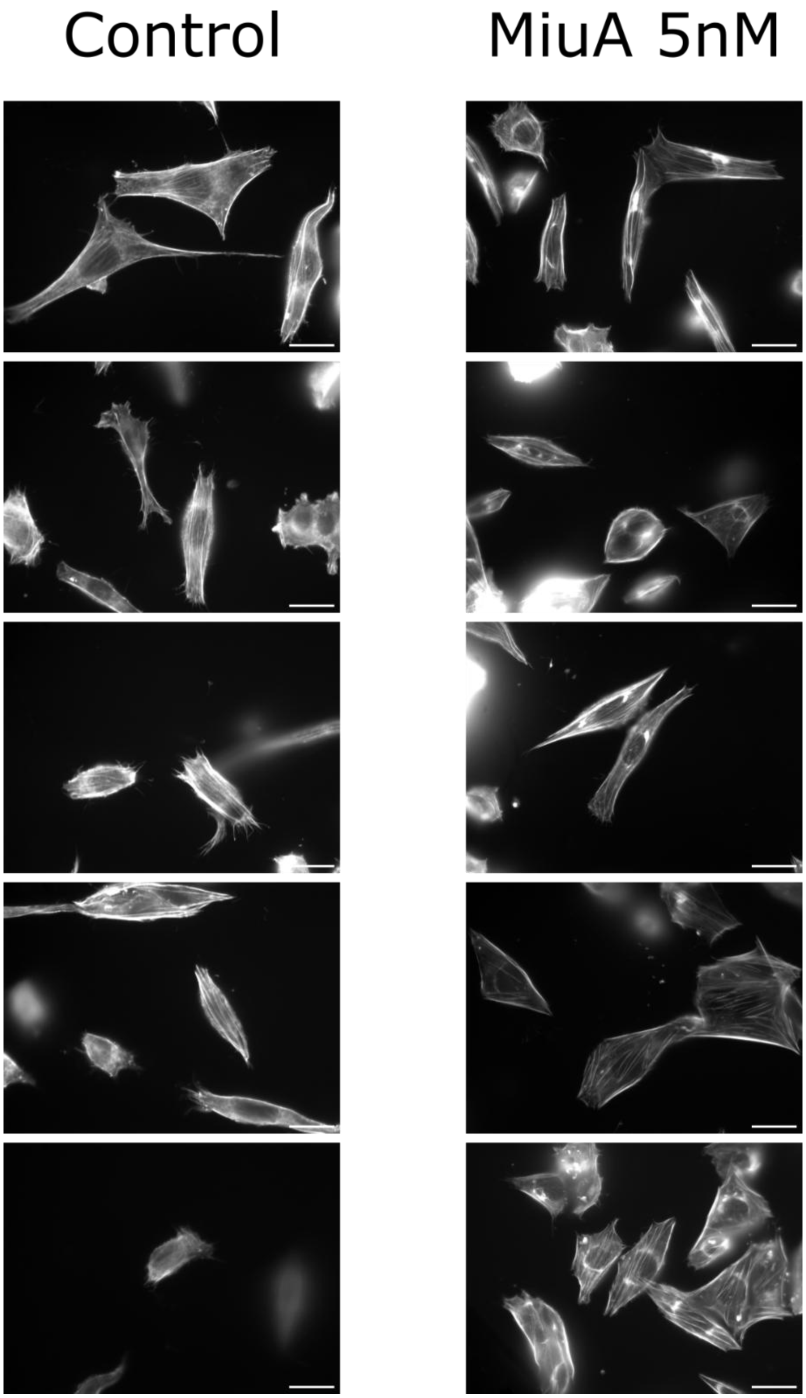

Fig S1. Additional images of MEF cells. Left: control, right: 5nM MiuA. Scale bar 20µm.
